# Social isolation during adolescence alters novel object recognition memory, brain and gut gene expression, and microbiota composition in a sex-specific manner

**DOI:** 10.1016/j.bbih.2026.101284

**Published:** 2026-06-08

**Authors:** Raise Ahmad, Gosia Zobel, Rina Hannaford, Paul Maclean, Trent Olson, Charlotte Hurst, Jeremy Bracegirdle, Wayne Young, Elizabeth Rettedal, Rachel C. Anderson, Julie E. Dalziel

**Affiliations:** a Bioeconomy Science Institute, Palmerston North, 4442, New Zealand; b Bioeconomy Science Institute, Hamilton, 3240, New Zealand

**Keywords:** (6-10 words) anxiety, Depression, Proximal colon, Immune, Microbiome, Myelin, Teenage

## Abstract

Adolescent social isolation is a known risk factor for anxiety and depression related disorders, yet its effects on brain–gut communication and potential sex differences remain unclear. We hypothesised that isolation would heighten anxiety- and depression-related behaviors and consequently impair memory in both sexes. To investigate this we exposed male (M) and female (F) rats to four weeks of social isolation beginning at 3 weeks of age and assessed behavior, brain and gut gene expression and microbiota, in single- (S) or pair-housed (P) animals. Contrary to our hypothesis, the results showed higher novel object recognition memory in socially-isolated females (FS vs FP). No isolation-induced changes in anxiety-related behaviours were detected in either sex. Social isolation in females (FS vs FP) increased expression of hippocampal *Grik5* (glutamate receptor/memory/learning), and decreased expression of prefrontal cortex genes: *Mbp*, *Mobp*, *Plp1* (neuroplasticity), *Cnp* (neuroprotection) and *Tph2* (serotonin synthesis). There was a trend toward lower microbial diversity in socially-isolated females (FS vs FP). Although no behaviour change was detected in isolated males, amygdala c-*Fos* (neuronal activity) and prefrontal cortex *Gabbr1* (inhibitory) expression were decreased. *Il6r*, *Tgfb1*, *Tlr9* (immune-related) were increased in the colon (MS vs MP). In both sexes, social isolation increased *Tph1* expression in the colon (FS vs FP; MS vs MP). These findings indicate sex-specific responses to adolescent social isolation, with females showing enhanced novel object recognition memory performance alongside changes in genes linked to neuroplasticity and memory, while males showed altered brain and gut gene expression linked to brain neuro-activity and gut-immune function.

## Highlights

• Social isolation improved female recognition memory and altered brain genes.
• Gut immune genes were increased in socially isolated males.
• Males were more anxious than females irrespective of housing.

## Graphical abstract

**Figure:**
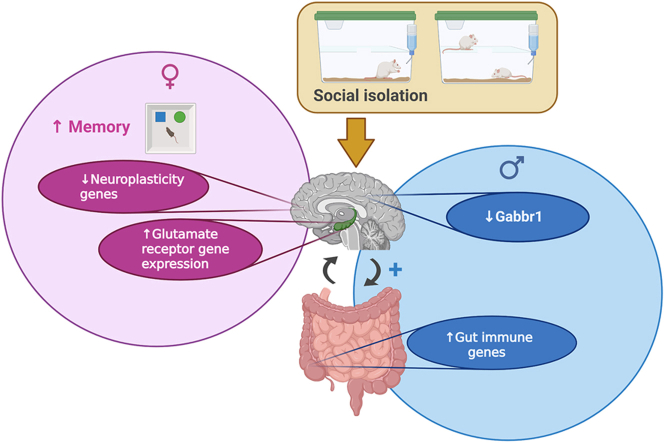
Created in BioRender. Dalziel, J. (2026) https://BioRender.com/p8qnh3p.

## 1 Introduction

Social isolation (SI) during adolescence is a significant environmental stressor associated with an increased risk of developing mood and cognitive disorders, including depression and anxiety (Brandt et al., 2022). This critical neurodevelopmental period of adolescence is marked by heightened brain plasticity and refinement of neural circuits that control emotion and cognition, and stress-induced disruptions can have lasting behavioral and neurobiological consequences (Andersen and Teicher, 2008; Romeo, 2017). Chronic stress experienced during adolescence can alter brain maturation trajectories, increasing susceptibility to psychiatric disorders (Lupien et al., 2009; Smith and Pollak, 2020).

In addition to anxiety- and depressive-like behaviours, adolescent social isolation has also been associated with increased impulsivity and risk-taking behaviours, further highlighting its broad impact on behavioral regulation (Bove et al., 2025; Liu et al., 2017; Venegas et al., 2025). Rodent models of post-weaning social isolation have been widely used to mimic chronic psychosocial stress during adolescence to induce anxiety-like and depressive behaviors, mainly in male rats. Following social isolation, they consistently show reduced exploration in open field and elevated plus maze tests (Lukkes et al., 2009; Weiss et al., 2004) reflecting anxiety, and impaired spatial (McCormick et al., 2012) and recognition memory (Bianchi et al., 2006) indicating cognitive deficits. At the neurochemical level, social isolation disrupts serotonergic (Soga et al., 2015), dopaminergic, and glutamatergic neurotransmission in male rats (Shao et al., 2015; Whitaker et al., 2013), along with dysregulation of the hypothalamic–pituitary–adrenal (HPA) axis which underlie the stress response (Lukkes et al., 2009). Furthermore, chronic social isolation in male mice has been shown to impair white matter integrity and neural connectivity in the prefrontal cortex, likely due to disrupted oligodendrocyte maturation and myelination processes (Liu et al., 2012; Makinodan et al., 2012) and induce structural changes at hippocampal synapses (Bianchi et al., 2006).

A gap in the social isolation literature is that only a few studies have included female rats (Walker et al., 2019). Where both sexes have been used to study social isolation during adolescence, the socially isolated males tend to be more anxious than females (Fone and Porkess, 2008; Weiss et al., 2004). These sex-dependent effects manifest as alterations in locomotor activity, anxiety- and depressive-like behaviours in the elevated plus maze, and reduced exploratory behaviour in the open field (Mumtaz et al., 2018; Xiong et al., 2023). Social isolation stress in mice during adolescence results in elevated aggressive behavior in stressed males and social withdrawal in females (Wang et al., 2022). Epidemiological data consistently show that females exhibit higher rates of stress-related disorders such as anxiety and depression (Kuehner, 2017) and preclinical evidence suggests female mice may demonstrate heightened behavioral and neuroendocrine sensitivity to social isolation (Senst et al., 2016). Differences in pubertal timing and developmental trajectories between sexes may further contribute to variability in behavioral and neurobiological responses to adolescent stress.

Another knowledge gap is that research to date has concentrated on brain outcomes, overlooking potential brain–gut–microbiota axis (BGA) pathways in stress-related changes in behavioral response and mental health consequences (Cryan et al., 2019; Foster et al., 2017). The BGA consists of complex bidirectional communication channels linking the central nervous system with the gastrointestinal tract through neural, immune, endocrine, and microbial pathways. Alterations in gut microbiota composition and gut barrier function have been linked to disruptions in brain processes, via immune modulation and production of neuroactive metabolites (Luczynski et al., 2016; Mayer et al., 2014; Sampson and Mazmanian, 2015). Social isolation has been shown to alter gut microbial composition and promote intestinal immune activation, although findings are variable and often sex-dependent (Bailey et al., 2011; Burokas et al., 2017). Importantly, few studies have examined these gut-related changes alongside concurrent behavioral and brain molecular outcomes within the same experimental framework.

To address these important knowledge gaps, we investigated the sex-specific effects of chronic social isolation during adolescence on behavior and gene expression changes within brain and gut. Sprague Dawley rats were selected as they are considered normo-sensitive because they exhibit lower baseline anxiety and stress responsivity compared with stress-sensitive strains such as Wistar Kyoto rats (Bassett et al., 2019), which display heightened HPA-axis reactivity (Fox et al., 2015). This allowed investigation of social isolation effects without a pre-existing high-stress phenotype. We hypothesised that social isolation would alter anxiety-related behaviours and memory, and that these changes may be accompanied by coordinated alterations in brain and gut-associated pathways. Specifically, we assessed behavioral outcomes (open field, elevated plus maze, and novel object recognition), gene expression in key brain regions (amygdala, hippocampus, and prefrontal cortex), and peripheral measures including gut gene expression and cecal microbiota composition to explore potential links across the brain–gut axis.

## 2 Methods

### 2.1 Animals

The research protocols used were approved by the AgResearch Animal Ethics Committee, New Zealand (AE1216-0370) in accordance with the Animal Welfare Act, 1999 (New Zealand). Sprague Dawley (SD) rats (24 male and 24 female) were from the same outbred breeding colony and the study carried out in two blocks of 24 rats at two different time points. They were weaned on postnatal day 18, transferred to the study location. Animals were from 7 litters and upon enrolment in the study (Day 0) were aged 19 post-natal days (PND) in block 1 and PND 19 in Block 2, bred as two separate cohorts. Rats were weighed (median, range: 42.6 g, 39.9 – 52.4 g male; 42.4 g, 38.8 – 50.6 g female) and then assigned to paired (2 rats/cage) or single (1 rat/cage) caging by semi-randomisation ensuring even weights across treatment groups. The rats were housed individually or in pairs in behavioral study double-decker rat green series cages (H-Temp-GR1800, Techniplast PTY Ltd. NHSW, Australia) providing a two-level, three-dimensional environment (462 mm × 390 mm x 243 h) with transparent walls. This cage meets ethical guideline standards (Branch, 2007) allowing a bipedal position to stretch up fully, along with jumping and exploring. Food (LabDiet - Prolab RMH, 1800) and water were provided *ad libitum*. The housing rooms were set to a 12 h/12 h reverse light–dark cycle with lights off at 0700 and ambient temperature of 19 ± 1 °C. Rats were weighed every seven days. Single-housed animals were physically separated but remained in the same room, allowing auditory and olfactory, but not physical, contact with other animals. Because social isolation stress can be reduced by exercise and environmental enrichment (Benite-Ribeiro et al., 2014), no exercise wheels were provided, nor was additional enrichment provided. Animals were acclimatised to the experimenter throughout, but handling was minimised using cylinders to transfer animals for cage cleaning and testing.

### 2.2 Study design

**Fig. 1:**
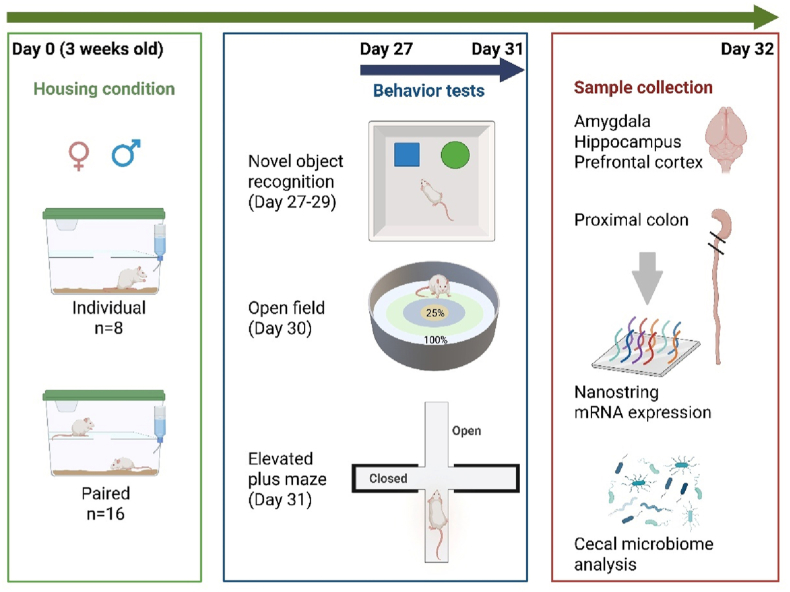
Study design. Created in BioRender. Dalziel, J. (2026) https://BioRender.com/6gno5jk.

A power analysis was used to estimate the required sample size for housing groups. The effect size was “large” at 80%, this corresponded with a minimum true difference of interest of 8.89 (18%) based on a SD of 8.7 based on rodent elevated plus maze (EPM) behavior (Savignac et al., 2014). The four treatment groups for housing and sex were: single females (FS, n = 8), paired females (FP, n = 16), single males (MS, n = 8), and paired males (MP, n = 16). The animals spent four weeks (Day 0 - 31) (PND 19-50) in either single or paired housing including four days of behavior testing (Fig. 1). The PND 21-49 equates to approximately 8-18 years of age in humans (Bell, 2018). The study was divided into two blocks that included all treatment groups and were offset by one week.

### 2.3 Behavioral analysis

The novel object recognition (NOR; Day 27 to Day 29), open field (OFT; Day 30), and the elevated plus maze (EPM; Day 31) tests were conducted as reported previously (Dalziel et al., 2023; Hurst et al., 2025). These behavioral assays were selected to assess exploratory behaviour, anxiety-related responses, and recognition memory.

### 2.4 Sample collection

Brain tissue samples of prefrontal cortex (PFC), hippocampus (HPC), and amygdala (AMG) regions, and proximal colon were collected immediately after euthanasia by carbon dioxide inhalation (Day 32). Tissue samples were stored in RNAlater (Ambion, Life Technologies, Carlsbad, CA) at −80 °C. Cecal contents were snap frozen in liquid nitrogen and stored at −80 °C for gut microbiota analysis.

### 2.5 Gene expression analysis

#### 2.5.1 RNA isolation

Total RNA from each brain region was extracted using RNeasy Lipid Tissue Mini Kits (QIAG74804, Qiagen, Auckland, New Zealand) and QIAzol Lysis Reagent from tissue (20 mg) homogenised using a TissueRuptor (Qiagen). RNA quality was assessed using an Agilent 2100 Bioanalyzer Instrument (Agilent, Santa Clara, CA, USA) and samples with an RNA integrity threshold >8 were used.

#### 2.5.2 mRNA analysis using nanostring

Gene expression analysis was carried out using the nCounter Analysis System (NanoString Technologies Inc., Seattle, WA) in conjunction with standard Elements TagSets chemistry protocols for a code set as reported previously (Dalziel et al., 2023). Custom designed rat gene-specific oligonucleotide reporter and capture probe pairs (Integrated DNA Technologies, PTE LTD, Singapore) and nCounter Elements™ TagSets (NanoString Technologies) were used. The code set consisted of probes for reference genes and probes specific for selected genes in brain (64) and proximal colon (31) based on their known or implicated involvement in brain receptor neurotransmission pathways associated with anxiety, depression, memory, neuroinflammation, neuroplasticity, and in the proximal colon inflammation and serotonergic transmission (Table 1, Supplementary Tables 1 and 2). Gene targets were selected based on prior evidence linking these pathways to stress responses, neuroplasticity and brain–gut interactions.

**Table 1:** Gene selection panel for brain and proximal colon.

Tissue	Functions	Gene symbol
Brain (Hippocampus amygdala, prefrontal cortex)	Neurotransmitters (GABA, glutamate, serotonin) in anxiety, depression, and memory	*Gabbr1, Gabbr2, Gabra1, Gabra2, Gabra4, Gabra5, Gabra6, Gabrb1, Gabrb2, Gabrg2, Slc6a12, Slc6a13, Gad1, Gad2, Gls, Glud1, Gria1, Gria3, Grik1, Grik2, Grik5, Grin1, Grin2a, Grin2b, Grin2c, Grm1, Grm2, Grm3, Slc17a7, Slc1a3, Htr1a, Htr1b, Htr1d, Htr4, Ht2c, Htr7, Slc6a4, Tph2*
	Neuroplasticity and memory	*Arc, Bdnf, c-Fos, Egr1, Crf, Cnp, Mbp, Mobp, Mtor, Ldha, Plp1, Snap25*
	Neuroimmune	*Cd200, Cd200r1, Creb1, Crh, Ifng, Il1a, Il1b, Il6, Il10, Il12b, Il17a, Nfkb1, Nos2, Tnf*
Proximal colon	Serotonin/tryptophan	*Ddc, Tph1, Htr1a, Htr1b, Htr2b, Htr2c*, *Htr4, Ido1, Kmo, Kynu, Maoa, Slc6a4*
	Immune response, inflammation, cytokine signalling	*Creb1, Cxcr1*, *Il1a, Il1b, Il2, Il4r, Il6, Il6r, Il10, Il10ra, Nfkb1, Socs3, Socs5, Tgfb1, Tlr2, Tlr4, Tlr9, Tnf, Nos2*

### 2.6 Microbiota

#### 2.6.1 DNA extraction and sequencing

Metagenomic DNA was extracted from cecal contents using Macherey Nagel Nucleospin Soil kits following the manufacturer's instructions (Dürren, Germany) and sequenced on an Illumina MiSeq as previously described (Ewels et al., 2016; Rettedal et al., 2019) using Massey Genome Service (Palmerston North, New Zealand).

#### 2.6.2 16S rRNA analysis

The 16S sequencing data was first quality checked using FastQC (Andrews, 2010) and MultiQC (add FastQC and MultiQC references here) to confirm high-quality read data. The data was then analysed using mothur following MiSeq SOP methods (Kozich et al., 2013; Rettedal et al., 2019). Paired-end reads were assembled, quality filtered (ambiguous bases, homopolymers >8 bp, Q25/50 bp), aligned to SILVA v.138 (Quast et al., 2012), pre-clustered (4 bp), and screened for chimeras using VSEARCH (Rognes et al., 2016).

After taxonomic classification, non-bacterial sequences were removed from the analysis and sequences were clustered into OTUs at a 97% cutoff. Taxonomic classification and count files were then exported from mothur for use in other data analysis programs.

MicrobiomeAnalyst was used to analyse and visualize taxonomic data (Lu et al., 2023). Taxonomic data were filtered to remove reads below a count of 5 (mean abundance value) and normalized using data rarefication to equal library size and total sum normalization (TSS). Alpha diversity was measured using the Shannon index (ANOVA, p ≤ 0.05). Beta diversity was measured using Bray Curtis principal coordinate analysis (PCoA) and a permutational multivariate analysis of variance (PERMANOVA) for statistical analysis (p ≤ 0.05). Permutational analysis of multivariate dispersions (PERMDISP) was performed to measure the homogeneity of group dispersions in complement to the PERMANOVA analysis. Linear discriminant analysis effect size (LEfSe) (Segata et al., 2011) analysis was performed to determine which taxa were significantly different between groups (linear discriminant analysis [LDA] ≥ 2, p ≤ 0.05) with an FDR correction. MaSaLin2 (Mallick et al., 2021) was used to do pairwise comparisons of treatment groups and determine statistically significant differences (default settings; p ≤ 0.05) with an FDR correction.

### 2.7 Statistical analysis

Graphs show boxplots of raw values, and model results are presented as predicted means and standard errors, unless otherwise stated. All data were analysed using R statistics software version v4.3.1, with data tidying using functions from the R packages tidyverse (Wickham et al., 2019), janitor (Firke, 2023), and data visualisation using the viridis colour palette (Garnier et al., 2023). Body weight was compared across treatment groups at week 1- 4 using repeated measures two-way ANOVA (Prism, GraphPad 10 software).

#### 2.7.1 Behavioral measures

A generalised linear model, with a Beta distribution for the response variable and a logit link function with the glmmTMB function from the glmmTMB package (Brooks et al., 2017) was fitted for the response in each of the behavior trials (OFT, EPM and NOR). All models included age at start of trial and block along with treatment (a combination of housing and sex) as fixed effects, and cage id was the random effect.

*Open field test (OFT).* The relative duration spent in each zone (25%, 50%, 75%, 100% + wall) was the response in addition to the fixed effects the model included zone as well as the main covariate of interest, and the zone/treatment interaction.

*Elevated plus maze test (EPM).* The response was the proportion of time spent in each zone of the EPM (close/open/centre) and included zone and the interaction between zone and treatment as fixed effects.

*Novel object recognition test (NOR).* The response variable was the recognition index, calculated as the time spent with the novel object divided by the total exploration time for both objects.

In all behavioral trials we also tracked the distance moved by each animal. For this data we fitted linear mixed models, fitted with the lmer function from the lme4 package (Bates et al., 2015) to assess movement based on the same fixed and random effects (excluding zone) as the Beta models. Due to a preference for the left side of the NOR arena, side preference was included in these models.

The models used were able to handle the imbalanced sample sizes and the residuals showed no issues (Pinheiro, 2014).

#### 2.7.2 Gene expression statistical analysis

Expression analysis was performed using Nanostring nCounter nSolver™ 4.0 (Nanostring MAN-C0019-08) with the Nanostring Advanced Analysis Module 2.0 plugin (Nanostring MAN-10030-03) while following the Nanostring gene expression data analysis guidelines (Nanostring MAN-C0011-04). The mRNA counts were generated in a tabulated form and were retrieved from the analyser as raw data (Reporter Code Count, RCC) files. The RCC files were imported into nSolver Analysis Software version 4 (https://www.nanostring.com/products/analysis-software/nsolver) for analysis. The software performed a quality control routine to flag samples for exclusion according to the following parameters: fields of view registration <75%; binding density outside the 0.05 to 2.25 range; positive control linearity: positive control R2 value < 0.95; and positive control limit of detection: 0.5 fM positive control ≤2 SD above the mean of the negative controls. All samples used for statistical analysis passed the quality control routine. Background subtraction was performed by subtracting the geometric mean of 6 internal negative controls with +3SD (standard deviation) from each sample. Positive control normalization was performed using the geometric mean of 6 internal positive controls to compute the normalization factor. The normalization factor of all samples was inside the 0.3 to 3 range. Reference gene normalization was performed using the geometric mean of counts for the 8 reference genes included in the ProbeSet (Supplementary Data 1). To compare different treatment groups permutation single factor ANOVA was performed as implemented in the lmPerm R package version 2.1.0 (Wheeler, 2010) on the normalized gene nanostring counts with post-hoc tests being performed with the predictmeans R package version 1.0.8 (Luo et al., 2018). Multiple testing correction was applied using the Benjamani-Hochberg false discovery rate method (Benjamini and Hochberg, 1995).

#### 2.7.3 Dataset integration

The multi-block sPLS-DA ‘DIABLO’ algorithm was implemented in the mixOmics R package version 6.28.0 with four treatment groups of combined housing and sex as the factor for discrimination. Overall, 239 features from across all 4 -omics types were used for analysis with DIABLO. The tune.block.spslda (Rohart et al., 2017) function was used to identify the optimal number of supervised features across each -omics type. A total of 28,561 models were fitted for each component, using the centroid.dist parameter with a 5-fold cross validation to identify the optimal number of features across behavior, gut gene, gut bacterial genera, and brain genes used for subsequent integration analysis downstream. Finally, these supervised features were fitted to block.splsda (Rohart et al., 2017) with an equally weighted design matrix contribution across -omics types that can discriminate and explain the phenotypic variance. There were two components used in this analysis.

## 3 Results

### 3.1 Body weight was unchanged by social isolation

There were no differences in percent body weight gain per week over the course of the study due to social isolation. Two-way ANOVA showed a significant interaction between time and sex (**F** (3) = 10.2, *P* < 0.0001), as well as significant main effects of time (**F** (3) = 730.9, *P* < 0.0001) and sex (**F** (1) = 138.8, *P* < 0.0001). There were no sex differences over days 1–14 (3–5 weeks of age), after which males and females diverged, gaining 47% and 34% over days 14–21, and 49% and 29% over days 21–28, respectively (Supplementary Fig. 1). Overall, body weight trajectories were not significantly affected by housing condition.

### 3.2 Behavior tests show no change in anxiety but improved novel object recognition in FS

Assessment of mood and anxiety-related behaviors (OFT and EPM) showed no significant effect due to social isolation, though prominent overall sex differences were observed. Memory in the NOR was affected by housing in a sex-dependent manner. Overall, social isolation did not significantly alter anxiety-related behaviors in either sex.

#### 3.2.1 Open field test

**Fig. 2:**
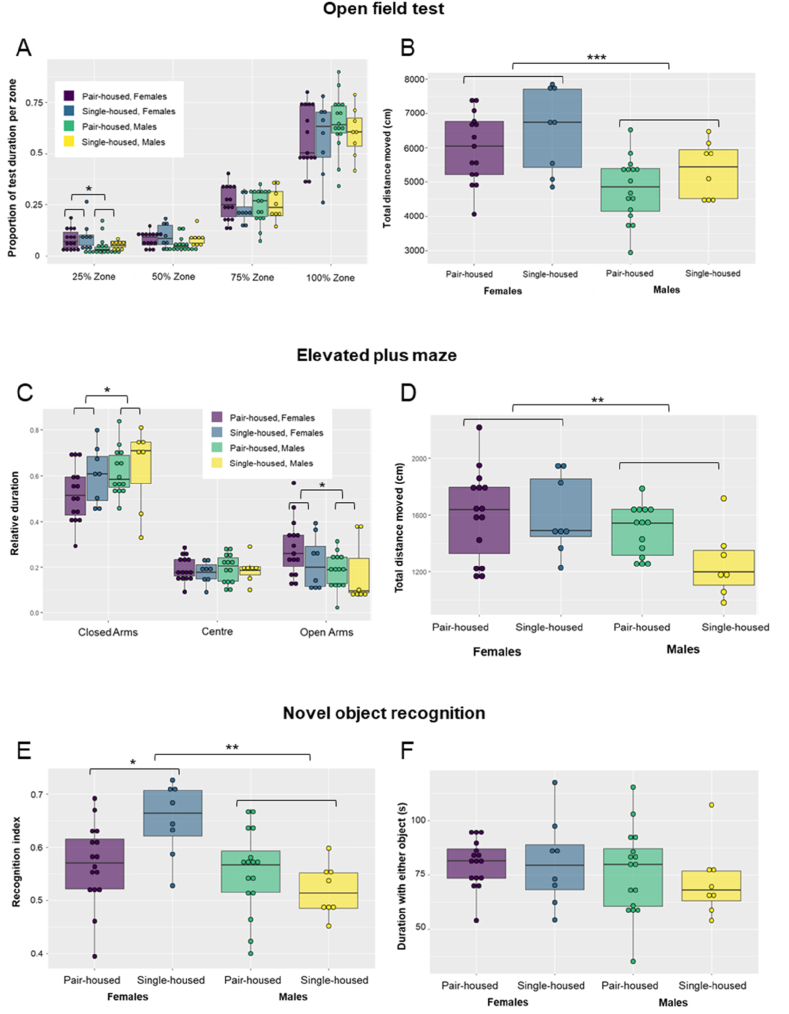
Effects of social isolation on behavior. No differences were found from social isolation in mood-related behaviors in open field (A&B) and elevated plus maze (C&D) tests. Social isolation induced a significant increase in recognition memory in females in the novel object recognition test (E&F). Paired females (n = 15-16), paired males (n = 15-16), single females (n = 7-8), single males (n = 7-8).

Housing treatment had no effect on proportion of test time that rats spent in the zones (P = 0.9), however there was interaction between sex and concentric zones of the arena for this variable (P = 0.017) (Fig. 2a). Males spent a lower proportion of time in the ‘25% (centre) zone’ than females (proportion of test time predicted in centre: 0.06 ± 0.01 vs. 0.09 ± 0.01, P = 0.05). There was strong sexual dimorphism for total distance moved during the test. Females travelled on average 1158 ± 280 cm more than males (P = 0.001) (Fig. 2b). Data was not used from one FP animal because it did not move in the arena. No significant main effect of housing was observed on locomotor or zone preference measures.

#### 3.2.2 Elevated plus maze

Overall, the rats spent proportionally more time in the closed arms (P = 0.0001). This was dependent on sex (P = 0.0001), with males spending more time in the closed arms than females (proportion of test time in closed arms: 0.67 vs. 0.56 ± 0.03, respectively, P = 0.02) (Fig. 2c). Correspondingly, males spent less time than females in the open arms (proportion of test time in closed arms: 0.16 ± 0.02 vs. 0.26 ± 0.03, respectively, P = 0.02). Only sex was significant for distance moved (P = 0.01). On average, male rats travelled 20% less than females (1317 ± 75 vs 1596 ± 72 cm), respectively, (P = 0.02), regardless of housing (Fig. 2d). There was a tendency for a three-way interaction between zone, sex, and housing treatment (P = 0.06). MP rats spent a higher proportion of time in the closed arms compared to FP rats (proportion of test time predicted in closed arms: 0.71 ± 0.03 vs. 0.52 ± 0.4, P = 0.0006). Correspondingly, MP rats spent less time in the open arms compared to FP rats (proportion of test time predicted in open arms: 0.15 ± 0.02 vs. 0.29 ± 0.03, P = 0.001). There was no difference between MP and MS in time spent in closed arms (P = 0.34) or time in open arms (P = 0.69). Data was not used from three animals across three treatment groups because they escaped from the EPM. There was no consistent main effect of social isolation on anxiety-like behavior measures.

#### 3.2.3 Novel object recognition

While this test does not assess mood, memory test performance can be impaired by increased anxiety or depression. Housing affected the recognition index (P = 0.007), and this relationship was dependent on sex (P = 0.004) (Fig. 2e). FS spent more time with the novel object compared to FP (R-index 0.57 ± 0.02 vs. 0.65 ± 0.02, P = 0.04). The higher recognition index for FS shows increased ability to discriminate between a new object and one previously encountered. The difference between MP and MS was not statistically significant (P = 0.51), this is likely driven by the high variation in the MP. MS rats spent less time with the novel object compared to FS (R-index 0.52 ± 0.02 vs. 0.65 ± 0.02, P = 0.002). Total duration of exploration with either object did not differ between groups (Fig. 2f). These findings indicate a sex-dependent effect of housing on novel object recognition performance, with a significant effect observed only in females.

### 3.3 Brain expression altered for myelin and neurotransmission genes in socially isolated females

#### 3.3.1 Regional differences in brain gene expression

The gene expression patterns across the three brain regions are shown for 54/65 genes expressed at detectable levels across all animals (Supplementary Fig. 2). The Cohen's d effect size for the gene expression data was calculated on fold changes and it averaged 0.58 for amygdala, 0.65 for hippocampus and prefrontal cortex and 1.1 for gut/colon, for each dataset indicating medium to large effect sizes. Seven immune-related genes and four neurotransmitter pathway genes were below detectable levels. Genes showed enriched expression in specific regions, for example: *Gabra5*, *Gria1*, *Grm1*, *Grin2a*, *Htr1b*, *Mobp*, *Mbp*, *Plp1*, *Cnp*, *Tph2* in HPC; *Grik1*, *Grik2*, *Il1a* in AMG; and *Grm2* and *Grm3* in PFC. These patterns reflect region-specific baseline gene expression differences.

#### 3.3.2 Social isolation

**Fig. 3:**
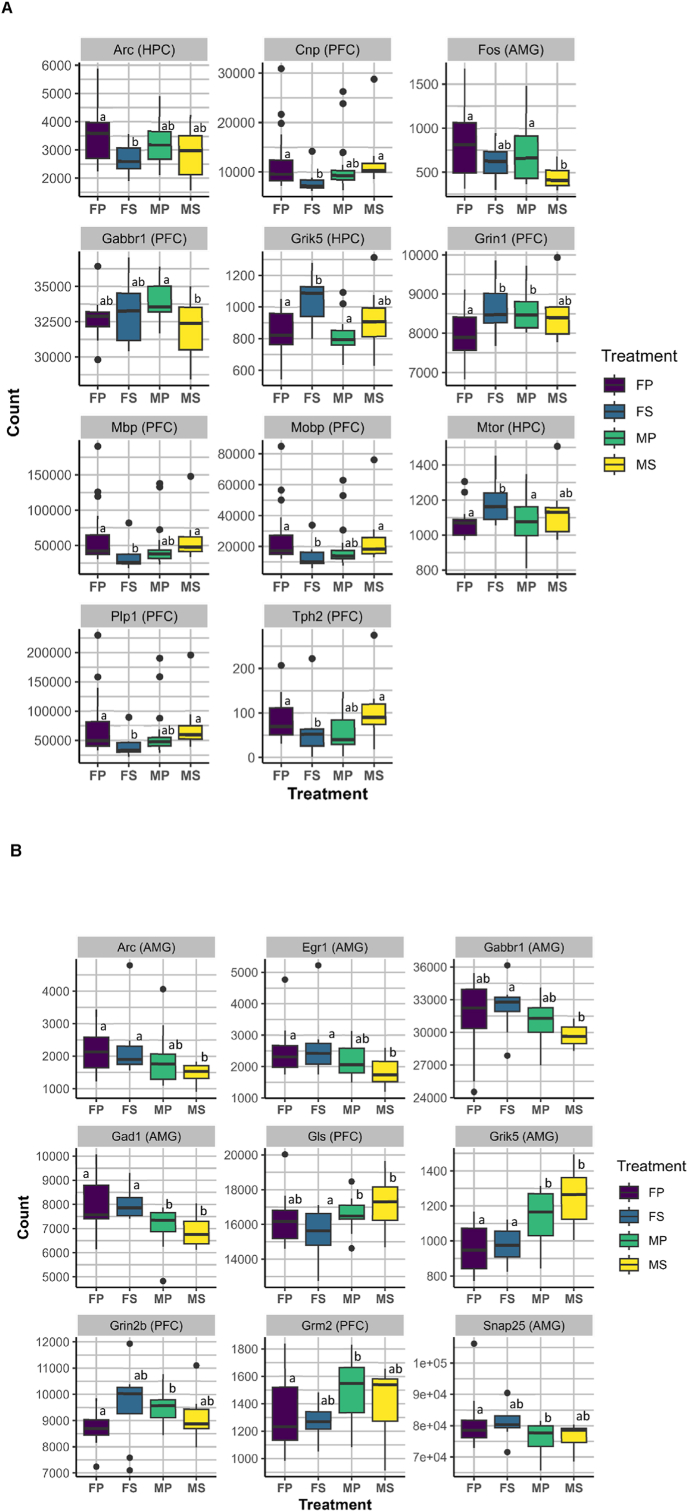
Differences in brain gene expression (mRNA copy number) due to A) social isolation, and B) sex differences, in amygdala (AMG), hippocampus (HPC), and prefrontal cortex (PFC) among treatment groups for paired females, FP (n = 16), single females, FS (n = 8), paired males, MP (n = 16), and single males, MS (n = 8). Analysis from post-hoc tests following permutation ANOVAs, non-overlapping letters indicate significant differences between treatments (post-hoc test p-value ≤0.05).

Social isolation-induced differences in brain gene expression in females were apparent for nine genes associated with synaptic plasticity, myelination, and neurotransmission in the HPC: *Arc* (↓25%), *Grik5* (↑25%), *Mtor* (↑11%); and in the PFC: *Cnp* (↓33%), *Grin1* (↑8%), *Mbp* (↓46%), *Mobp* (↓48%), *Plp1* (↓46%), *Tph2* (↓27%) (Fig. 3a, Supplementary Table 3). In comparison, only two brain genes were altered by social isolation in males (AMG: *Fos* ↓38% and PFC: *Gabbr1* ↓6%). Six immune related brain genes were unchanged with social isolation in either males or females (*Cd200, Cd200r1, Creb1, Crh, Il1a, Nfkb1*). Overall, gene expression changes due to social isolation were more pronounced in females than males.

#### 3.3.3 Sex differences

Thirteen different genes showed sex differences (Fig. 3b). Two genes were altered in expression in the AMG; *Gad1* was decreased in males compared with females, whereas *Grik5* was increased in males compared to females, under both housing conditions. Ten genes showed sex differences for individually housed animals, notably PFC levels were elevated for *Mobp*, *Plp1*, *Tph2* in MS compared with FS. These results indicate strong sex-dependent baseline differences in brain gene expression.

### 3.4 Proximal colon gene expression altered for immune function in socially isolated males

#### 3.4.1 Peripheral changes in gene expression

To assess gut changes in gene expression with social isolation, we assessed tissue expression in the proximal colon (Supplementary Fig. 3) because this region is innervated by vagal afferents and is a likely site of active host-microbiome interactions due to its cecal proximity.

#### 3.4.2 Social isolation

**Fig. 4:**
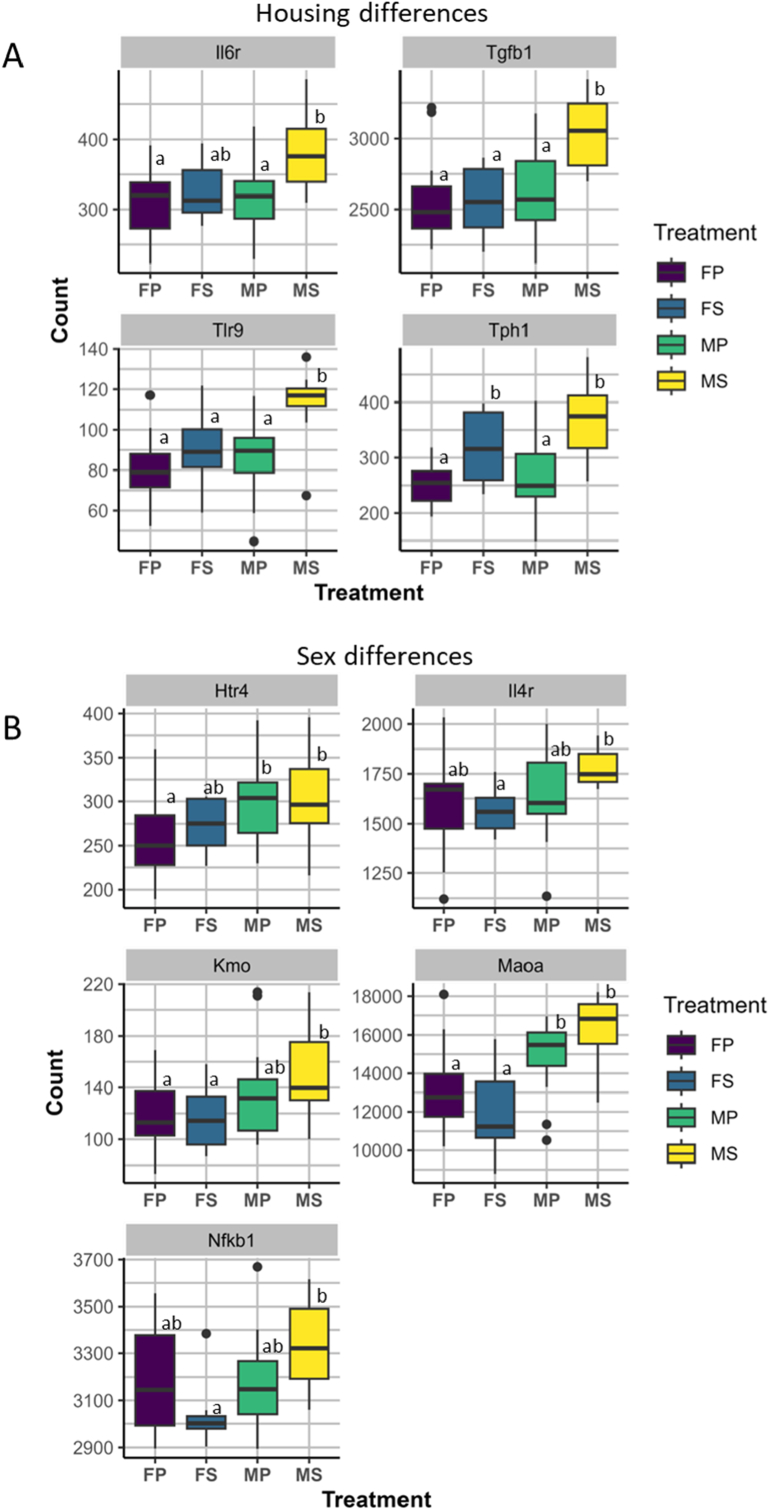
Differences in proximal colon gene expression (mRNA copy number) due to social isolation (A) and sex differences (B), among treatment groups for paired females, FP (n = 16), single females, FS (n = 8), paired males, MP (n = 16), and single males, MS (n = 8). Analysis from post-hoc tests following permutation ANOVAs, non-overlapping letters indicate significant differences between treatments (post-hoc test p-value ≤0.05).

*Tph1* was increased by social isolation in both males (39%) and females (27%) and is the gene that encodes tryptophan hydroxylase 1, the rate-limiting enzyme in the synthesis of serotonin in peripheral tissues (Fig. 4a, Supplementary Table 4). Three genes were increased in colonic expression in MS: *Il6r* (21%), *Tgfb1* (16%), and *Tlr9* (30%) which are associated with immune functions.

#### 3.4.3 Sex differences

Five genes showed only sex differences in gut gene expression (Fig. 4b). One gene in particular (*Maoa*), was higher in males than in females for individual and pair housed. *Ilr4* and *Nfkb* genes were higher in MS than FS. Of those associated with serotonin pathways, *Htr4* expression (5-HT4 serotonin receptor) was higher in PM than PF, and *Kmo* expression (kynurenine 3-monooxygenase enzyme (KMO) involved in tryptophan metabolism) was higher in MS than FS. Overall, sex differences were more pronounced than housing effects in proximal colon gene expression.

### 3.5 Microbiome changes were driven by sex differences rather than social isolation with small changes for socially isolated females

**Fig. 5:**
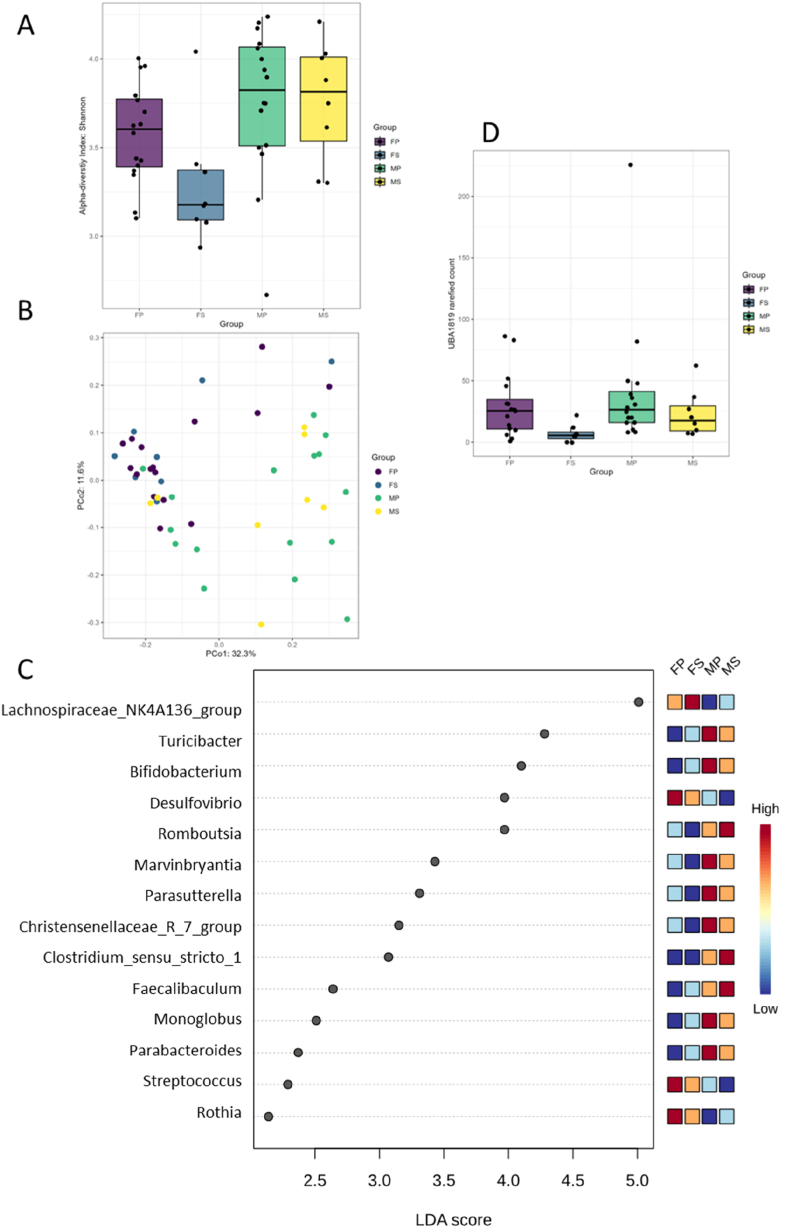
Cecal microbiome composition indicates differences were mainly due to sex rather than social isolation for: (A) alpha-diversity (Shannon Index; p-value: 0.015814; [ANOVA] F-value: 3.8419) and (B) beta-diversity (OTU level; PERMANOVA F-value: 3.0959; R-squared: 0.17429; p-value: 0.001). (C) LEFSE analysis at a genus level identified significantly different taxa between treatment groups, scale on right indicates relative abundance within taxa. (D) Genus identified as significantly different in MaAsLin2 pairwise comparison of FS vs FP groups (Log2FC = 2.19, p-value FDR = 0.0419).

To further explore possible gut-brain impacts of social isolation, we assessed the cecal microbiome (Supplementary Fig. 4). Alpha diversity analyses indicated a significantly lower diversity (p-value FDR ≤0.05) in FS compared to both male groups and a trend towards lower diversity compared to the FP group (p-value = 0.06) (Fig. 5a, Supplementary Table 5). The PCoA plot indicated a separation between males and females but no separation based on social isolation status (Fig. 5b) and PERMANOVA analysis showed a significant difference between groups (p-value ≤0.05). Pairwise group PERMANOVA comparisons (p-value FDR ≤0.05) highlight that sex was the primary contributor (Supplementary Table 6). A PERMDISP analysis was not significant (p-value = 0.1932), indicating the PERMANOVA results were not due to greater dispersion between groups. LeFSe analysis at genus level identified taxa that were significantly different between groups (p-value FDR ≤0.05; LDA ≥2; Fig. 5c). Further analyses indicated differences were primarily due to sex with housing status having little to no significant difference within the same sex (Supplemental Tables 7 and 8). The only identified significant difference due to housing status was the uncharacterised *Ruminococcaceae*, UBA1819, which was lower in FS compared to FP (Fig. 5d).

### 3.6 Dataset integration revealed BGA links between anxiety, gut genes, and microbiota

**Fig. 6:**
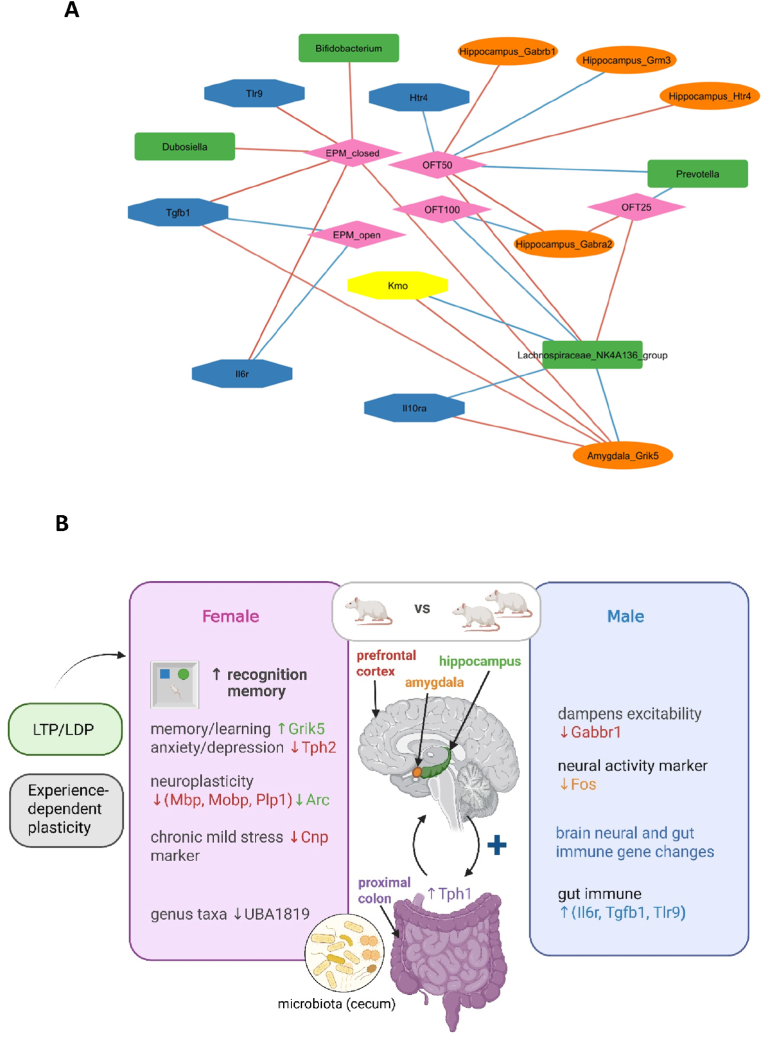
Dataset associations identified by the multi-block sPLS-DA ‘DIABLO’ algorithm implemented in the mixOmics R package displayed as (A) a network plot of the associations among datasets where the nodes are annotated by datasets as follows: behavioral test parameters (pink diamonds), brain genes (orange ellipses), proximal colon genes (blue octagons), cecal bacteria (green rounded rectangles). Variables with an association score >0.65 are joined by a red line and variables with an association score < −0.65 are joined by a blue line. (B) Model of brain-gut axis changes with social isolation. LTP (long term potentiation), LDP (long term depression). Created in BioRender. Dalziel, J. (2026) https://BioRender.com/sz8rmfu. (For interpretation of the references to colour in this figure legend, the reader is referred to the Web version of this article.)

Behavioral parameters associated with mood were identified that associated with changes across the other three datasets (Fig. 6a, Supplementary Fig. 5). For example, EPM closed time (where higher reflects increased anxiety), was positively associated with Grik5 (AMG), *Tgfb1*, *Tlr9*, *Il6r* (proximal colon), and cecal microbiota *Bifidobacterium* and *Dubiosiella*. *Tgfb1*, *Tlr9*, and *Il6r* were also all identified as being increased in expression by social isolation in the proximal colon in males. In addition, the OFT100 (time in the outer zone, which when higher may reflect reduced willingness to explore the centre reflecting increased anxiety) was negatively associated with *Gabra2* (HPC) and cecal *Lachnospiraceae* NK4A136 group. In contrast, OFT 25/50 (higher reflects less anxiety), were variously associated with HPC: *Gabbr1* (+), *Gabra2* (+), *Grm3* (−), *Htr4* (+), proximal colon *Htr4* (−), and cecal microbiota *Lachnospiraceae* NK4A136 group (+), *Prevotella* (−). Notably there were no strong associations between memory and brain neuroplasticity genes detected with integration analysis. This is likely because while these seven genes were largely decreased in females by social isolation, half of these showed much greater sex differences, adding complexity to the relationships that was not resolved by the algorithm. BGA changes in relation to social isolation are summarised according to sex differences (Fig. 6b).

## 4 Discussion

Our main finding in this adolescent social isolation model was a female-specific improvement in novel object recognition memory, accompanied by alterations in brain–gut axis (BGA) function, without changes in anxiety-related behaviours in either males or females. This contrasts with our hypothesis that social isolation would primarily alter mood and thus impair memory. The female-specific response suggests that social isolation may have acted as a mild environmental challenge and/or experience dependent stimulus, influencing novel object recognition performance in a sex-dependent manner. Strong sex differences in behavioral measures, gene expression, and microbiota were also observed, largely independent of housing.

Improved novel object recognition observed in FS is a novel finding specific to performance in the NOR task, reflecting greater discrimination between new and familiar objects. In contrast, socially isolated males showed the lowest recognition index and minimal object exploration. Lower baseline anxiety in females may have contributed to this sexual dimorphism. In males, previous studies of 30–33 days of adolescent social isolation report either no memory change or memory deficits, indicating context-dependent impairments in recognition memory (Bianchi et al., 2006; Nádeníček et al., 2022). In females, adolescent social isolation has been reported to impair novel object recognition and attentional performance, with rapid memory decay and reduced discrimination under similar paradigms (McLean et al., 2010), while partial or enriched social housing conditions can rescue these deficits across both sexes (Lodha et al., 2023). Furthermore, early-life social isolation suppresses neuroplasticity-related signalling and impairs memory formation in both males and females (Liu et al., 2020), supporting a general vulnerability of cognitive processes to social deprivation. Importantly, these effects are strongly dependent on developmental timing, as isolation during adulthood does not consistently reproduce recognition memory deficits observed during adolescence (Medendorp et al., 2018; Sullens et al., 2021).

Our results show that females spent more time in the OFT centre and EPM open arms than males indicating lower anxiety-like behaviour in females. However, social isolation did not significantly alter anxiety-related behaviours in the present study. Females also travelled greater distances than males in both tests, consistent with previously reported sex differences in locomotor activity. Distances travelled were comparable to our previous studies in MS (Bassett et al., 2019), MP and FP (Parkar et al., 2024). It remains unclear why social isolation did not alter anxiety-related behaviours in our study, as single-housed adolescent males have previously shown reduced exploration in OFT and EPM compared with paired or grouped animals (Dunphy-Doherty et al., 2018; Matsumoto et al., 2019; Novoa et al., 2021; Weiss et al., 2004). Although EPM medians for time spent in closed arms trended in the expected direction, they did not reach statistical significance.

Although social isolation–induced anxiety is well reported, behavioral responses vary depending on duration and developmental stage (Fan and Pedersen, 2021; Fone and Porkess, 2008; Lukkes et al., 2009). For example, 2–3 weeks of isolation induces anxiogenic or depressive-like behaviours in males (Dunphy-Doherty et al., 2018; Matsumoto et al., 2021; Novoa et al., 2021), whereas longer duration may produce fewer overt anxiety changes but subtle effects such as altered open-arm entries (Weiss et al., 2004) or increased locomotion interpreted as hyperactivity suggesting environmental adaptation (Dunphy-Doherty et al., 2018). Isolation beginning in early adolescence (P21–28) is anxiogenic in males (Parker and Morinan, 1986; Weiss et al., 2004), whereas later adolescent isolation may be anxiolytic (Einon and Morgan, 1977). The female-specific effects on memory and gene expression observed here may therefore be developmentally related, as females enter puberty earlier than males, and alignment by pubertal stage rather than chronological age may better resolve sex-dependent effects (Fone and Porkess, 2008).

Our data demonstrate that novel object recognition was increased in FS and brain gene expression altered in regions important for memory (HPC) and cognition (PFC). These changes may reflect coordinated relationships between behavior and molecular profiles, but do not address causality. The main shifts in brain gene expression due to social isolation in females involved genes implicated in synaptic plasticity and neurotransmission (*Grik5, Grin1*, *Mtor*) and myelination *(Mbp*, *Mobp*, *Plp1*, *Arc*). Because NOR is hippocampal-dependent, performance relates to synaptic plasticity.

Memory processes involve long-term potentiation (LTP) and long-term depression (LTD), which regulate synaptic strength. Kainate-type ionotropic glutamate receptors in the HPC contribute to these processes (Contractor et al., 2001), and thus we speculate that increased expression of *Grik5* (*GluK5, KA2*) in FS might be consistent with altered glutamatergic signalling. Similarly, altered *Grin1* expression suggests potential involvement of plasticity-related pathways (Lee et al., 2003). Elevated *Mtor* expression in the HPC also aligns with its involvement in memory formation. Altered *Tph2* expression in the PFC in FS may reflect dysregulated serotonergic signaling commonly observed following social isolation (Walker et al., 2019). Collectively, these coordinated changes in glutamatergic and serotonergic pathways provide a mechanistic framework for the female-specific enhancement of memory following adolescent social isolation (Walker et al., 2019).

Altered hippocampal *Arc* expression in FS is notable given its role in synaptic plasticity; however, *Arc* is also highly context and region-dependent. Changes in PFC myelin-related genes (*Mbp*, *Mobp, Plp1*) may suggest alterations in oligodendrocyte-associated transcription. This may reflect region-specific or developmental-stage dependent myelin remodeling rather than altered neural function. Importantly, the absence of changes in neuroimmune gene expression in the brain suggests that the observed molecular alterations are not likely to be associated with overt neuroinflammatory responses. Therefore, the present findings likely reflect subtle adaptive neuroplastic responses rather than pathological stress effects. The reduction in *Cnp* expression in the PFC of FS is also notable, as C-type natriuretic peptide (CNP) has been implicated in neuroprotection and stress-associated signalling (Ahmed et al., 2024).

Reduced *c-Fos* expression in the amygdala (AMG) of socially isolated adolescent males is consistent with region and sex-dependent neural activation changes following social isolation (Ahern et al., 2016). In MS, increased proximal colon expression of *Tlr9*, *Il6r*, *Tgfb1*, and *Tph1* infers altered immune and serotonergic signalling in the gut. Elevated *Tlr9* suggests enhanced microbial sensing, while increased *Tgfb1* and *Il6r* may reflect activation of immunoregulatory pathways and compensatory maintenance of epithelial integrity (Guo et al., 2021). Upregulation of *Tph1* could enhance serotonin-mediated signalling in enterochromaffin cells, promoting cytokine release and potentially contributing to gut dysbiosis (Shajib et al., 2017). Notably, proximal colon *Tph1* was also increased in FS, which could elevate gut serotonin, accelerate colonic transit via the enteric nervous system, and alter microbiota, although such microbial changes were not observed in either FS or MS, suggesting additional modulatory factors.

Differences in cecal microbiota composition were primarily shaped by sex rather than social isolation. The alpha diversity showed no significant change in microbial diversity due to social isolation; however, there was a trend towards lower diversity in FS vs FP. Beta diversity analyses consistently indicated that sex was the dominant driver of microbial separation. Differential abundance analyses identified several genera that differed between treatment groups, with sex emerging as the primary driver of these differences. The only taxon significantly affected by social isolation was UBA1819 (*Ruminococcaceae*), which was reduced in FS (Lopizzo et al., 2021). Sex-specific gut microbiome differences have frequently been observed highlighting the need to assess the impacts of sex independently (del Castillo-Izquierdo et al., 2022; Hokanson et al., 2024; Jašarević et al., 2016; Jiang et al., 2020; Oppenheimer et al., 2025).

Dataset integration revealed correlations between behavior, gene expression and microbiome suggesting coordinated but partially independent adaptations across brain and gut systems in response to adolescent housing condition. Dataset integration showed a positive association of *Dubosiella* and *Bifidobacterium* with time spent in the EPM closed arms which may suggest these taxa were associated with increased anxiety. Many studies have also shown an association with *Bifidobacterium* and beneficial effects on mental health (Huang and Liu, 2024; Li et al., 2023; Tamayo et al., 2025) and some *Bifidobacterium* species can produce gamma aminobutyric acid (GABA), an important inhibitory neurotransmitter (Duranti et al., 2020). *Lachnospiraceae* NK4A136 had multiple associations with both anxiety behavior and gene expression data, with greater abundance suggesting less anxiety and downregulation of inflammatory response genes in the gut and a gene involved in amygdala glutamate binding. Associations between cecal *Bifidobacterium* and *Lachnospiraceae* NK4A136 group with anxiety-related behaviors were also found in our previous social isolation study in the stress sensitive Wistar Kyoto rat strain (Hurst et al., 2025).

Overall, our study demonstrates adolescence as a key developmental window during which environmental housing conditions produced sex-dependent effects on behaviour and neurobiology. This mirrors human studies showing that adolescent social environment influences cognitive development and prefrontal cortex function (Wong et al., 2022). The rat adolescent social isolation model may provide a useful experimental paradigm for studying the neurodevelopmental consequences of reduced social interaction during adolescence, a critical period for social and cognitive maturation.

### 4.1 Limitations and future perspectives

While behavioral assessment was restricted to a specific set of test paradigms, future studies incorporating broader behavioral testing, longitudinal designs, and functional approaches will be important to establish underlying mechanisms. The inherent nature of comparing single versus paired animals created challenges in study design with the most suitable choice resulting in unequal group numbers. However, appropriate statistical models were used to account for this design structure. Targeted mRNA gene expression profiling does not capture genome-wide changes or provide protein-level or functional validation, however it does capture low level copy number for low level gene expression such as neurotransmitter receptors. Integrative analyses such as DIABLO help highlight potential connections between datasets, but they are typically based on correlations which do not represent proof of specific biological activities. These associations suggest potential interactions to explore in future studies.

## 5 Conclusion

Our study demonstrates that males and females respond differently to social isolation during adolescence. Our new discovery of improved recognition memory in socially isolated adolescent females alongside shifts in genes associated with neuroplasticity-related processes, learning, and memory suggests sex-specific neural responses. This may suggest an adaptive ability of the female brain in responding to environmental stress through enhanced cognitive performance. In males, changes in gut immune-related gene expression support peripheral physiological adaptation associated with social isolation. The higher level of anxiety in males compared with females and their different microbiome compositions highlight distinct sex differences in phenotype at a system level. The sexual dimorphism evident across multiple measures demonstrates the necessity of including both sexes in stress studies, especially during adolescence. Overall, the observed effects suggest coordinated but distinct sex-specific adaptations to adolescent social environment rather than uniform stress-induced phenotypes.

## Ethics statement

The animal study protocol was approved by the AgResearch Animal Ethics Committee (Hamilton, New Zealand; AEC approval number AE1216/0370) and carried out in accordance with the Animal Welfare Act, 1999 (New Zealand).

## Funding

This work was supported by the Endeavour Research Programme “Smarter Lives: New opportunities for dairy products across the lifespan” C10X1706 from the New Zealand Ministry of Business, Innovation and Employment.

## CRediT authorship contribution statement

**Raise Ahmad:** Conceptualization, Data curation, Investigation, Methodology, Writing – original draft, Writing – review & editing. **Gosia Zobel:** Conceptualization, Investigation, Writing – original draft. **Rina Hannaford:** Formal analysis. **Paul Maclean:** Formal analysis. **Trent Olson:** Formal analysis, Methodology. **Charlotte Hurst:** Formal analysis, Methodology. **Jeremy Bracegirdle:** Formal analysis, Methodology. **Wayne Young:** Conceptualization, Funding acquisition, Supervision, Writing – review & editing. **Elizabeth Rettedal:** Formal analysis, Writing – original draft, Writing – review & editing. **Rachel C. Anderson:** Conceptualization, Funding acquisition, Project administration, Supervision, Writing – review & editing. **Julie E. Dalziel:** Conceptualization, Data curation, Funding acquisition, Investigation, Supervision, Writing – original draft, Writing – review & editing.

## Declaration of competing interest

The authors declare that they have no known competing financial interests or personal relationships that could have appeared to influence the work reported in this paper.

## Data Availability

The original data presented in the study are openly available upon reasonable request from the Bioeconomy Science Institute. We have deposited our microbiome data at NCBI bioproject ID: PRJNA1455333.

## Appendix A Supplementary data

Supplementary Tables (8) “Supplementary Tables.docx”

Supplementary Figures (5) “Supplementary Figures.pptx”

Supplementary Data “Supplementary Data.xlsx”
