# Supplementary material for: Social isolation during adolescence alters novel object recognition memory, brain and gut gene expression, and microbiota composition in a sex-specific manner
Source: Brain Behav Immun Health. 2026 Jun 8;55:101284. doi: 10.1016/j.bbih.2026.101284 (PMC13272542; doi:10.1016/j.bbih.2026.101284)
Supplement: Multimedia component 1 [file mmc1.docx]

**Supplementary Table 1.** Brain gene expression

| **Gene** | **Name** | **Accession #** |
| --- | --- | --- |
| *Arc* | activity-regulated cytoskeleton-associated protein | NM_019361.1 |
| *Bdnf* | brain-derived neurotrophic factor | NM_001270630.1 |
| *Cd200* | Cd200 molecule | NM_031518.1 |
| *Cd200r1* | CD200 receptor 1 | NM_023953.1 |
| *Cnp* | metabolism | NM_012809.1 |
| *Creb1* | cAMP responsive element binding protein 1 | NM_134443.2 |
| *Crh* | corticotropin releasing hormone | NM_031019.1 |
| *Egr1* | early growth response 1 | NM_012551.2 |
| *Fos* | transcription factor, synaptic connections | NM_022197.2 |
| *Gabbr1* | metabotropic GABA receptor – inhibitory signalling | NM_031028.3 |
| *Gabbr2* | Gamma-Aminobutyric Acid Type B Receptor Subunit 2 | NM_031802.2 |
| *Gabra1* | Gamma-Aminobutyric Acid Type A Receptor Subunit alpha1 | NM_183326.2 |
| *Gabra2* | Gamma-Aminobutyric Acid Type A Receptor Subunit alpha2 | NM_001135779.1 |
| *Gabra4* | Gamma-Aminobutyric Acid Type A Receptor Subunit alpha4 | NM_080587.3 |
| *Gabra5* | Gamma-Aminobutyric Acid Type A Receptor Subunit alpha5 | NM_017295.1 |
| *Gabra6* | Gamma-Aminobutyric Acid Type A Receptor Subunit alpha6 | NM_021841.1 |
| *Gabrb1* | gamma-aminobutyric acid type A receptor subunit beta1 | NM_012956.1 |
| *Gabrb2* | gamma-aminobutyric acid type A receptor subunit beta3 | NM_012957.2 |
| *Gabrg2* | gamma-aminobutyric acid type A receptor subunit gamma2 | NM_183327.1 |
| *Gad1* | L-glutamate decarboxylase 1 (cytoplasmic GABA) | NM_017007.1 |
| *Gad2* | L-glutamate decarboxylase 2 (vesicular pool GABA) | NM_012563.1 |
| *Gls* | glutaminase (GLS1) brain - nerve terminal, mitochondria | NM_001109968.3 |
| *Glud1* | L-glutamate dehydrogenase - mitochondria | NM_012570.1 |
| *Gria1* | Glutamate Ionotropic Receptor AMPA Type (Subunit 1) | NM_031608.1 |
| *Gria3* | Glutamate Ionotropic Receptor AMPA Type Subunit 3 | NM_032990.2 |
| *Grik1* | Glutamate Ionotropic Receptor Kainate Type Subunit 1 (isoform 3) | NM_001111114.1 |
| *Grik2* | Glutamate Ionotropic Receptor Kainate Type Subunit 2 | NM_019309.2 |
| *Grik5* | glutamate ionotropic receptor kainate type subunit 5 | NM_031508.2 |
| *Grin1* | glutamate ionotropic receptor NMDA type subunit 1 | NM_001270602.1 |
| *Grin2a* | Glutamate Ionotropic Receptor NMDA Type Subunit 2A | NM_012573.3 |
| *Grin2b* | Glutamate Ionotropic Receptor NMDA Type Subunit 2B | NM_012574.1 |
| *Grin2c* | Glutamate Ionotropic Receptor NMDA Type Subunit 2C | NM_012575.3 |
| *Grm1* | Glutamate Metabotropic Receptor 1 | NM_017011.1 |
| *Grm2* | glutamate metabotropic receptor 2 | NM_001105711.1 |
| *Grm3* | Metabotropic glutamate receptor 3 | NM_001105712.1 |
| *Htr1a* | 5-Hydroxytryptamine Receptor 1A | NM_012585.2 |
| *Htr1b* | 5-Hydroxytryptamine Receptor 1B | NM_022225.2 |
| *Htr1d* | 5-Hydroxytryptamine Receptor 1D | NM_012852.1 |
| *Htr2c* | 5-Hydroxytryptamine Receptor 2C | NM_012765.3 |
| *Htr4* | 5-Hydroxytryptamine Receptor 4 | NM_012853.2 |
| *Htr7* | 5-Hydroxytryptamine Receptor 7 | NM_022938.2 |
| *Ifng* | interferon gamma | NM_138880.2 |
| *Il1a* | Interleukin 1a | NM_017019.2 |
| *Il1b* | Interleukin 1b | NM_031512.2 |
| *Il6* | Interleukin 6 | NM_012589.2 |
| *Il10* | interleukin 10 | NM_012854.2 |
| *Il12b* | interleukin 12B | NM_022611.2 |
| *Il17a* | interleukin 17A | NM_001106897.1 |
| *Itgam* | integrin subunit alpha M | NM_012711.1 |
| *Ldha* | lactate dehydrogenase A | NM_017025.1 |
| *Mbp* | myelination | NM_001025291.1 |
| *Mobp* | myelination | NM_001389273.1 |
| *Mtor* | mechanistic target of rapamycin kinase | NM_019906.1 |
| *Nfkb1* | nuclear transcription factor kappa B subunit 1 | NM_001276711.2 |
| *Nos2* | nitric oxide synthase 2 | NM_012611.3 |
| *Plp1* | myelination | NM_030990.1 |
| *Slc1a2 (Eaat2)* | solute carrier family 1 member 2 (glutamate transporter) |  |
| *Slc1a3 (Eaat1)* | solute carrier family 1 (glutamate transporter) | NM_019225.1 |
| *Slc6a4* | solute carrier family 6 member 4 | NM_013034.4 |
| *Slc6a12* | solute carrier family 6 member 12 | NM_017335.1 |
| *Slc6a13* | solute carrier family 6 member 13 | NM_133623.1 |
| *Slc17a7* | solute carrier family 17 | NM_053859.1 |
| *Snap25* | synaptosome associated protein 25 | NM_030991.3 |
| *Tnf* | tumor necrosis factor | NM_012675.3 |
| *Tph2* | serotonin synthesis and neurotransmission/signalling | NM_173839.2 |

**Supplementary Table 2.** Proximal colon gene expression

| **Gene** | **Name** | **Accession #** |
| --- | --- | --- |
| *Creb1* | cAMP responsive element binding protein 1 | NM_134443.2 |
| *Cxcr1* | C-X-C motif chemokine receptor 1 | NM_019310.2 |
| *Ddc* | dopa decarboxylase, transcript variant 1 | NM_012545.4 |
| *Htr1a* | 5-Hydroxytryptamine Receptor 1A | NM_012585.2 |
| *Htr1b* | 5-Hydroxytryptamine Receptor 1B | NM_022225.2 |
| *Htr2b* | 5-Hydroxytryptamine Receptor 2B | NM_017250.2 |
| *Htr2c* | 5-Hydroxytryptamine Receptor 2C | NM_012765.3 |
| *Htr4* | 5-Hydroxytryptamine Receptor 4 | NM_012853.2 |
| *Ido1* | indoleamine 2,3-dioxygenase 1 | NM_023973.2 |
| *Il10* | Interleukin 10 | NM_012854.2 |
| *Il10ra* | Interleukin 10 receptor | NM_057193.2 |
| *Il1a* | Interleukin 1A | NM_017019.2 |
| *Il1b* | Interleukin 1B | NM_031512.2 |
| *Il2* | Interleukin 2 | NM_053836.1 |
| *Il4r* | Interleukin 4 receptor | NM_133380.2 |
| *Il6* | Interleukin 6 | NM_012589.2 |
| *Il6r* | Interleukin 6 receptor | NM_017020.3 |
| *Kmo* | kynurenine 3-monooxygenase | NM_021593.2 |
| *Kynu* | kynureninase | NM_053902.2 |
| *Maoa* | monoamine oxidase A | NM_033653.1 |
| *Nfkb1* | nuclear transcription factor kappa B subunit 1 | NM_001276711.2 |
| *Nos2* | nitric oxide synthase 2 | NM_012611.3 |
| *Slc6a4* | solute carrier family 6 member 4 | NM_013034.4 |
| *Socs3* | suppressor of cytokine signaling 3 | NM_053565.1 |
| *Socs5* | suppressor of cytokine signaling 5 | NM_001109274.1 |
| *Tgfb1* | transforming growth factor, beta 1 | NM_021578.2 |
| *Tlr2* | toll-like receptor 2 | NM_198769.2 |
| *Tlr4* | toll-like receptor 4 | NM_019178.2 |
| *Tlr9* | toll-like receptor 9 | NM_198131.1 |
| *Tnf* | tumor necrosis factor | NM_012675.3 |
| *Tph1* | Tryptophan hydroxylase 1 | NM_001100634.3 |

**Supplementary Table 3.** Brain gene expression differences with housing and sex

|  |  | **Housing differences** | |  | **Sex differences** | |  |  |
| --- | --- | --- | --- | --- | --- | --- | --- | --- |
| **Gene** | **PF** | **IF (IF/PF)** ^a^ | **PM** | **IM (IM/PM)** ^a^ | **PM/PF** ^a^ | **IM/IF** ^a^ | **F-statistic (df=3)** | **p-value** |
| **AMG** |  |  |  |  |  |  |  |  |
| *Arc* | 2188 ± 44 | 2295 ± 132 | 1909 ± 50 | 1487 ± 39 | - | ↓35% | 2.590 | 0.063 |
| *Egr1* | 2464 ± 46 | 2673 ± 138 | 2202 ± 35 | 1852 ± 66 | - | ↓31% | 2.747 | 0.053 |
| *Fos* | 836 ± 25 | 624 ± 27 | 711 ± 21 | 444 ± 17 (↓38%) | - | - | 2.914 | 0.045 |
| *Gabbr1* | 31529 ± 206 | 32335 ± 308 | 30965 ± 119 | 29702 ± 130 | - | ↓8% | 1.480 | 0.230 |
| *Gad1* | 7894 ± 64 | 8033 ± 83 | 7154 ± 49 | 6858 ± 84 | ↓9% | ↓15% | 4.459 | 0.007 |
| *Grik5* | 959 ± 8.4 | 976 ± 13 | 1133 ± 9 | 1250 ± 22 | ­18% | ­28% | 9.208 | <0.001 |
| *Snap25* | 80780 ± 484 | 80872 ± 668 | 76364 ± 282 | 76492 ± 544 | ­6% |  | 2.328 | 0.082 |
| **HPC** |  |  |  |  |  |  |  |  |
| *Arc* | 3616 ± 273 | 2696 ± 215 (↓25%) | 3251 ± 197 | 2881 ± 325 | - | - | 2.331 | 0.086 |
| *Grik5* | 839 ± 36 | 1047 ± 55 (­25%) | 815 ± 29 | 926 ± 73 | - | - | 4.415 | 0.008 |
| *Mtor* | 1076 ± 23 | 1195 ± 51 (­↑11%) | 1077 ± 34 | 1140 ± 59 | - | - | 2.001 | 0.124 |
| **PFC** |  |  |  |  |  |  |  |  |
| *Cnp* | 12300 ± 1673 | 8173 ± 916 (↓33%) | 11228 ± 1415 | 12863 ± 2317 | - | ­57% | 1.827 | 0.152 |
| *Gabbr1* | 32780 ± 346 | 33154 ± 825 | 33994 ± 355 | 31984 ± 785 (↓6%) | - | - | 2.787 | 0.051 |
| *Gls* | 16180 ± 351 | 15497 ± 525 | 16617 ± 218 | 17213 ± 544 | - | ­11% | 2.775 | 0.052 |
| *Grin1* | 7967 ± 153 | 8626 ± 241 (­8%) | 8581 ± 139 | 8464 ± 245 | - | - | 3.627 | 0.019 |
| *Grin2b* | 8782 ± 166 | 9639 ± 554 | 9545 ± 149 | 9147 ± 331 | ­9% | - | 2.372 | 0.082 |
| *Grm2* | 1316 ± 64 | 1280 ± 48 | 1493 ± 57 | 1410 ± 93 | ­13% | - | 2.027 | 0.124 |
| *Mbp* | 63234 ± 11344 | 34056 ± 7303 (↓46%) | 50547 ± 8753 | 61257 ± 13082 | - | - | 2.535 | 0.067 |
| *Mobp* | 26695 ± 5145 | 13823 ± 3198 (↓48%) | 20172 ± 3937 | 26499 ± 7381 | - | ­92% | 2.452 | 0.074 |
| *Plp1* | 75695 ± 14140 | 40833 ± 7625 (↓46%) | 64413 ± 11323 | 77577 ± 17833 | - | ­90% | 2.221 | 0.096 |
| *Tph2* | 85 ± 12 | 62 ± 24 | 58 ± 11 | 107 ± 27 | - | ­73% | 2.478 | 0.067 |
|  |  | (↓27%) |  |  |  |  |  |  |

Mean ± SEM. ^a^ Percent change is shown for significantly different treatment groups for social isolation. Nanostring RNA gene expression analysis using nSolver software followed by permutation single factor ANOVA. Treatment effects are shown for genes that were altered in expression by individual compared with paired housing, and sex differences. Significance is from permutation analysis. HPC; hippocampus, AMG; amygdala, PFC; prefrontal cortex.

Housing differences in green. Sex differences in orange.

**Supplementary Table 4.** Proximal colon gene expression

| **Gene** | **PF** | **IF**  **(IF/PF)^a^** | **PM** | **IM**  **(IM/PM)^a^** | **F-statistic**  **(df=3)** | **p-value** |
| --- | --- | --- | --- | --- | --- | --- |
| *Htr4* | 257 ± 11 | 273 ± 11 | 297 ± 12 | 303 ± 20 | 2.246 | 0.042 |
| *Il4r* | 1617 ± 59 | 1563 ± 40 | 1633 ± 54 | 1781 ± 34 | 0.586 | 0.172 |
| *Il6r* | 307 ± 12 | 327 ± 16 | 315 ± 12 | 382 ± 21  (↑21%) | 1.314 | 0.013 |
| *Kmo* | 119 ± 7 | 117 ± 9 | 135 ± 9 | 151 ± 13 | 2.207 | 0.101 |
| *Maoa* | 13030 ± 517 | 11997 ± 903 | 14945 ± 469 | 16366 ± 669 | 5.671 | <0.001 |
| *Nfkb1* | 3167 ± 56 | 3041 ± 52 | 3171 ± 50 | 3335 ± 69 | 2.517 | 0.043 |
| *Tgfb1* | 2555 ± 76 | 2559 ± 88 | 2620 ± 82 | 3042 ± 98 (↑16%) | 2.665 | 0.005 |
| *Tlr9* | 81 ± 4 | 91 ± 7 | 87 ± 4 | 112 ± 7 (↑30%) | 2.563 | 0.009 |
| *Tph1* | 251 ± 9 | 318 ± 24 (↑27%) | 264 ± 16 | 369 ± 25 (↑39%) | 2.698 | <0.001 |

Mean ± SEM. ^a^ Percent change is shown for significantly different treatment groups for social isolation. Nanostring RNA gene expression analysis using nSolver software followed by permutation single factor ANOVA. Treatment effects are shown for genes that were altered in expression by individual compared with paired housing, and sex differences. Significance is from permutation analysis. Green shaded are housing differences and orange show sex differences.

**Supplementary Table 5.** Shannon diversity index ANOVA group pairwise comparison.

| Pair | Statistic | P-value | FDR |
| --- | --- | --- | --- |
| FP vs MP | -1.35 | 0.19 | 0.27 |
| FP vs FS | 2.14 | 0.06 | 0.11 |
| FP vs MS | -1.28 | 0.22 | 0.27 |
| MP vs FS | 2.9 | 0.01 | 0.04 |
| MP vs MS | 0.06 | 0.95 | 0.95 |
| FS vs MS | -2.85 | 0.01 | 0.04 |

**Supplementary Table 6.** Beta diversity PERMANOVA group pairwise comparison.

| Pair | F-value | R-squared | P-value | FDR |
| --- | --- | --- | --- | --- |
| FP vs MP | 5.69721 | 0.159598 | 0.001 | 0.006 |
| FP vs FS | 1.12142 | 0.048501 | 0.316 | 0.3792 |
| FP vs MS | 3.65621 | 0.142508 | 0.005 | 0.01 |
| MP vs FS | 3.96365 | 0.152662 | 0.003 | 0.009 |
| MP vs MS | 0.677347 | 0.029869 | 0.767 | 0.767 |
| FS vs MS | 2.5954 | 0.156393 | 0.016 | 0.024 |

**Supplementary Table 7.** MaAsLin2 pairwise comparisons between all groups.

| **FS vs MS** | **Log2FC** | **St.Error** | **P-value** | **FDR** |
| --- | --- | --- | --- | --- |
| Bifidobacterium | -4.47 | 1.22 | 0.000682 | 0.0356 |
| Candidatus_Saccharimonas | -4.54 | 1.19 | 0.000414 | 0.0356 |
| Turicibacter | -4.9 | 1.34 | 0.000707 | 0.0356 |
| **FS vs MP** | **Log2FC** | **St.Error** | **P-value** | **FDR** |
| Turicibacter | -5.57 | 1.16 | 1.96E-05 | 0.00202 |
| Corynebacterium | -2.6 | 0.57 | 3.99E-05 | 0.00246 |
| Marvinbryantia | -4.41 | 1.03 | 0.000103 | 0.00418 |
| Bifidobacterium | -4.33 | 1.06 | 0.000183 | 0.00627 |
| UBA1819 | -2.63 | 0.695 | 0.00046 | 0.0108 |
| Parasutterella | -2.15 | 0.591 | 0.000717 | 0.0148 |
| Odoribacter | -1.99 | 0.559 | 0.000888 | 0.0163 |
| Christensenellaceae_R_7_group | -3.07 | 0.878 | 0.00109 | 0.0179 |
| Romboutsia | -3.42 | 0.978 | 0.0011 | 0.0179 |
| Acetatifactor | -2.14 | 0.632 | 0.00148 | 0.0217 |
| Clostridium_sensu_stricto_1 | -4.03 | 1.2 | 0.00161 | 0.0217 |
| Faecalibaculum | -3.29 | 0.976 | 0.00159 | 0.0217 |
| Monoglobus | -2.69 | 0.828 | 0.0022 | 0.0272 |
| UCG_010_ge | -2.59 | 0.793 | 0.00215 | 0.0272 |
| UCG_009 | -2.64 | 0.816 | 0.00233 | 0.0276 |
| Elusimicrobium | -2.33 | 0.74 | 0.00298 | 0.0318 |
| Blautia | -3.79 | 1.22 | 0.00333 | 0.0332 |
| Candidatus_Saccharimonas | -3.2 | 1.03 | 0.00327 | 0.0332 |
| Bacteroidia_unclassified | -1.9 | 0.633 | 0.00436 | 0.0393 |
| **MP vs MS** | No significant differences | | | |
| **FP vs MS** | **Log2FC** | **St.Error** | **P-value** | **FDR** |
| Streptococcus | 1.44 | 0.336 | 9.45E-05 | 0.0292 |
| Bifidobacterium | -3.82 | 1.06 | 0.000787 | 0.0356 |
| Clostridium_sensu_stricto_1 | -4.31 | 1.2 | 0.00082 | 0.0356 |
| Eggerthellaceae_unclassified | 2.64 | 0.744 | 0.000922 | 0.0356 |
| Turicibacter | -4.22 | 1.16 | 0.000744 | 0.0356 |
| Faecalibaculum | -3.39 | 0.976 | 0.00118 | 0.0404 |
| **FP vs MP** | **Log2FC** | **St.Error** | **P-value** | **FDR** |
| Turicibacter | -4.89 | 0.95 | 5.91E-06 | 0.00183 |
| Corynebacterium | -2.26 | 0.466 | 1.56E-05 | 0.00202 |
| Streptococcus | 1.28 | 0.274 | 2.92E-05 | 0.00226 |
| Faecalibaculum | -3.58 | 0.797 | 5.04E-05 | 0.0026 |
| Bifidobacterium | -3.68 | 0.866 | 0.000108 | 0.00418 |
| Clostridium_sensu_stricto_1 | -3.87 | 0.979 | 0.000277 | 0.00857 |
| Marvinbryantia | -3.29 | 0.843 | 0.000316 | 0.00889 |
| Monoglobus | -2.59 | 0.676 | 0.000396 | 0.0102 |
| Parabacteroides | -1.43 | 0.381 | 0.000488 | 0.0108 |
| Acetatifactor | -1.84 | 0.516 | 0.000898 | 0.0163 |
| Barnesiella | -2.01 | 0.586 | 0.00132 | 0.0204 |
| Parasutterella | -1.55 | 0.483 | 0.00241 | 0.0276 |
| Rothia | 1.46 | 0.457 | 0.0026 | 0.0286 |
| Dubosiella | -2.76 | 0.921 | 0.00445 | 0.0393 |
| Prevotella | -2.78 | 0.922 | 0.00428 | 0.0393 |
| **FP vs FS** | **Log2FC** | **St.Error** | **P-value** | **FDR** |
| UBA1819 | 2.19 | 0.695 | 0.0029 | 0.0419 |

**Supplementary Table 8.** LeFSe comparison between female and male rats.

| **Females vs Males** | **Pvalues** | **FDR** | **F** | **M** | **LDAscore** |
| --- | --- | --- | --- | --- | --- |
| Lachnospiraceae_NK4A136_group | 0.00028446 | 0.0025045 | 358360 | 204060 | 4.89 |
| Lachnospiraceae_unclassified | 0.014969 | 0.04896 | 114480 | 87395 | 4.13 |
| Desulfovibrio | 0.00026257 | 0.0025045 | 27033 | 13044 | 3.84 |
| Butyricicoccus | 0.012226 | 0.041975 | 2881.1 | 1424.3 | 2.86 |
| Streptococcus | 8.4582e-05 | 0.0017424 | 559.42 | 256.59 | 2.18 |
| Rothia | 0.00029178 | 0.0025045 | 481.92 | 243.57 | 2.08 |
| UCG_010_ge | 0.011325 | 0.041662 | 120.48 | 324.32 | -2.01 |
| Odoribacter | 0.0019678 | 0.012668 | 231.19 | 439.59 | -2.02 |
| Parabacteroides | 0.00061775 | 0.0048945 | 367.95 | 741.12 | -2.27 |
| Monoglobus | 0.00026211 | 0.0025045 | 119.18 | 602.4 | -2.38 |
| Muribaculaceae_unclassified | 0.0088083 | 0.03629 | 689.02 | 1343.5 | -2.52 |
| Anaerostipes | 0.0084163 | 0.03612 | 0.65125 | 697.48 | -2.54 |
| Faecalibaculum | 3.2878e-05 | 0.0008466 | 215.56 | 1066.1 | -2.63 |
| Allobaculum | 0.015736 | 0.04896 | 379.02 | 1829.3 | -2.86 |
| Candidatus_Saccharimonas | 0.017112 | 0.04896 | 618.68 | 2093.8 | -2.87 |
| Blautia | 0.0092023 | 0.036455 | 623.24 | 2244.8 | -2.91 |
| Clostridium_sensu_stricto_1 | 2.8141e-06 | 0.00014493 | 1.9537 | 1656.8 | -2.92 |
| Christensenellaceae_R_7_group | 0.011835 | 0.041975 | 980.12 | 2642.1 | -2.92 |
| Parasutterella | 0.00083554 | 0.0061472 | 2151.1 | 4717.6 | -3.11 |
| UCG_008 | 0.01661 | 0.04896 | 743.07 | 3589.7 | -3.15 |
| Clostridia_UCG_014_ge | 0.015395 | 0.04896 | 2394 | 5363.7 | -3.17 |
| Dubosiella | 0.0011617 | 0.0079769 | 1438.6 | 4487.1 | -3.18 |
| Marvinbryantia | 0.00011473 | 0.0019695 | 758.05 | 4799.7 | -3.31 |
| UCG_005 | 0.0073452 | 0.03612 | 1450.3 | 7360.4 | -3.47 |
| Romboutsia | 0.00028435 | 0.0025045 | 11043 | 24462 | -3.83 |
| Bifidobacterium | 8.4346e-06 | 0.00028959 | 6074.2 | 28392 | -4.05 |
| Turicibacter | 2.8216e-07 | 2.9062e-05 | 4577.6 | 38714 | -4.23 |
| Prevotella | 0.0078156 | 0.03612 | 34815 | 104490 | -4.54 |
