# Supplementary material for: Social isolation during adolescence alters novel object recognition memory, brain and gut gene expression, and microbiota composition in a sex-specific manner
Source: Brain Behav Immun Health. 2026 Jun 8;55:101284. doi: 10.1016/j.bbih.2026.101284 (PMC13272542; doi:10.1016/j.bbih.2026.101284)
Supplement: Multimedia component 3 [file mmc3.pptx]

## Slide 1
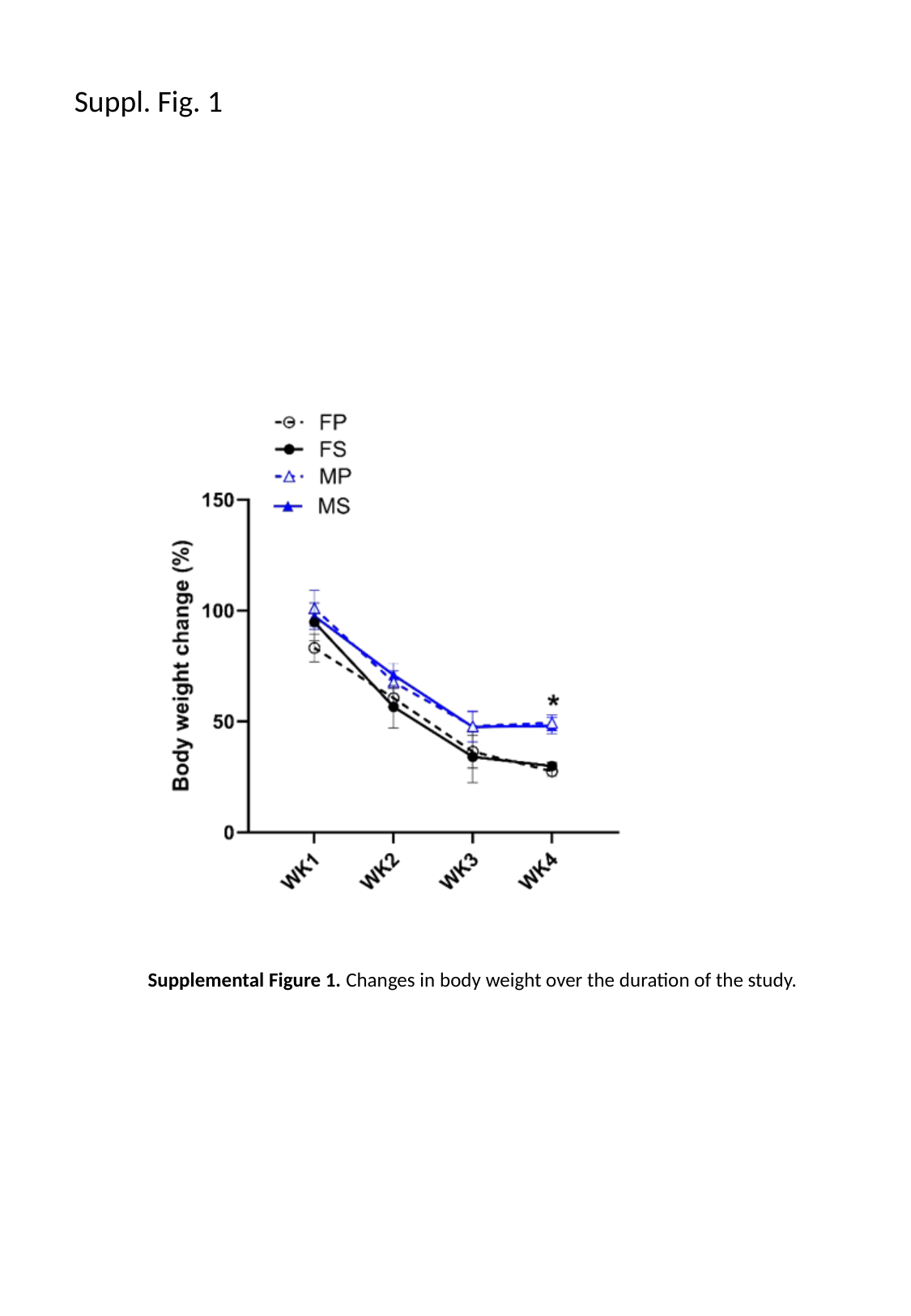

Suppl. Fig. 1
Supplemental Figure 1. Changes in body weight over the duration of the study.

## Slide 2
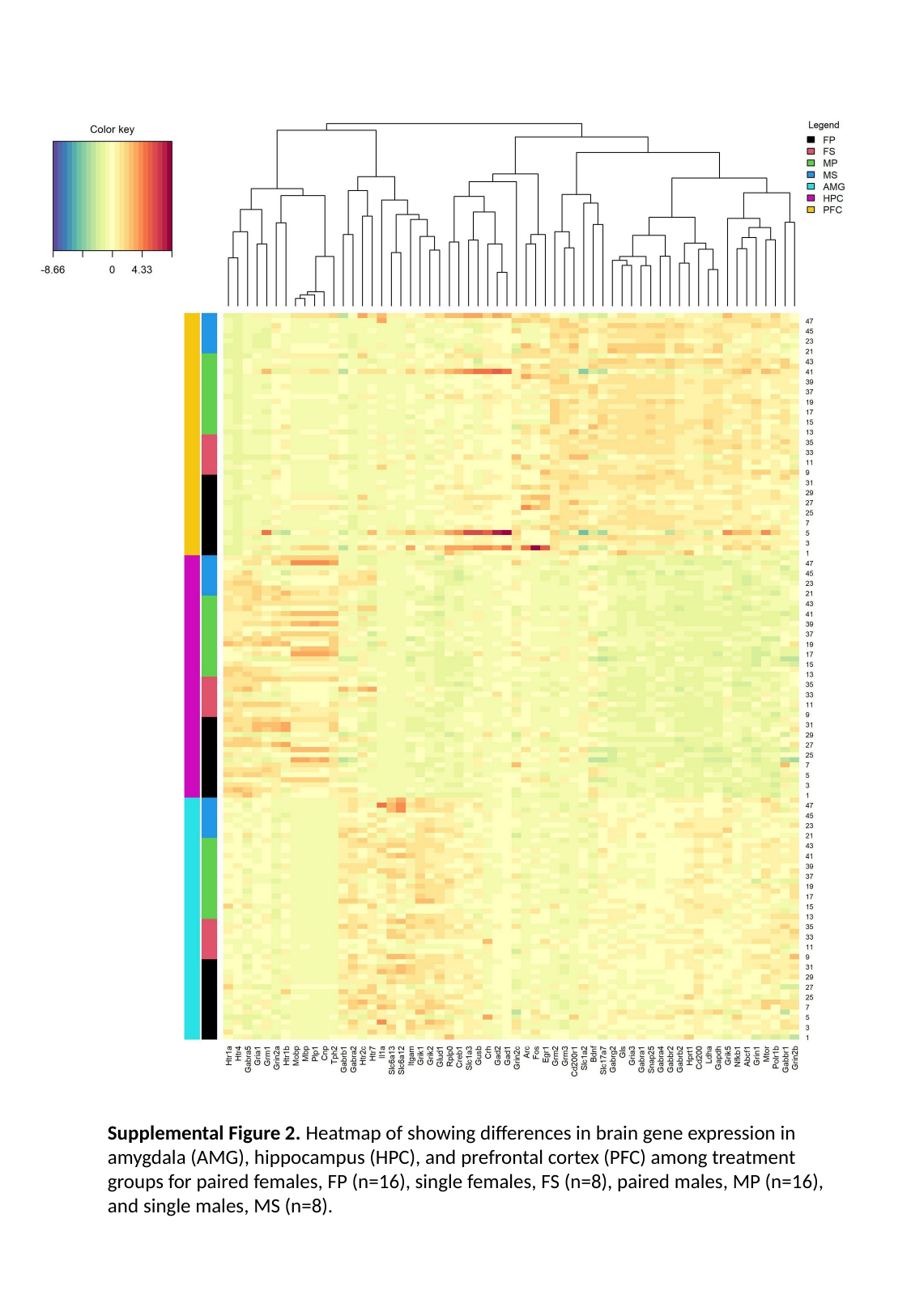

Supplemental Figure 2. Heatmap of showing differences in brain gene expression in amygdala (AMG), hippocampus (HPC), and prefrontal cortex (PFC) among treatment groups for paired females, FP (n=16), single females, FS (n=8), paired males, MP (n=16), and single males, MS (n=8).

## Slide 3
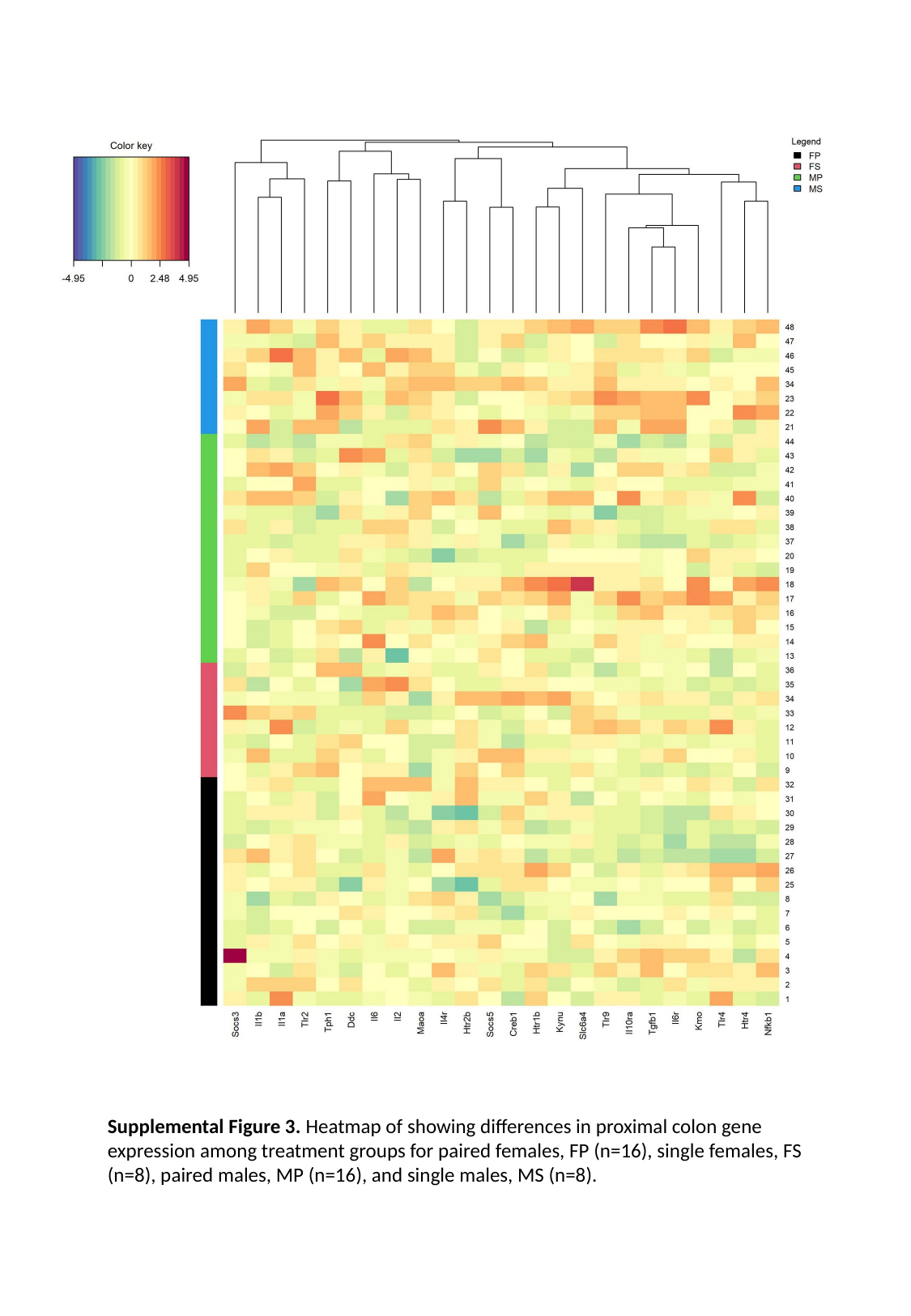

Supplemental Figure 3. Heatmap of showing differences in proximal colon gene expression among treatment groups for paired females, FP (n=16), single females, FS (n=8), paired males, MP (n=16), and single males, MS (n=8).

## Slide 4
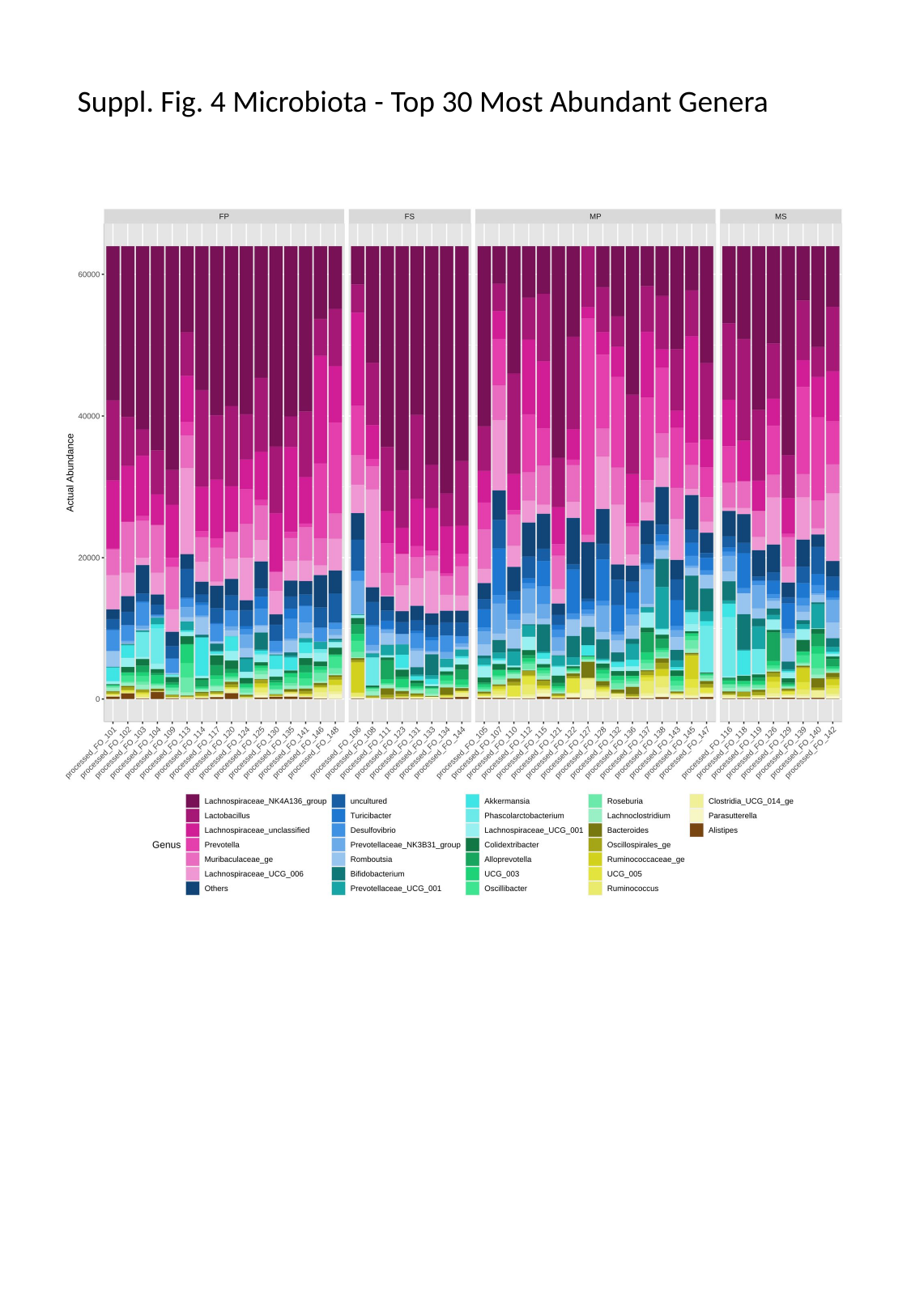

Suppl. Fig. 4 Microbiota - Top 30 Most Abundant Genera

## Slide 5
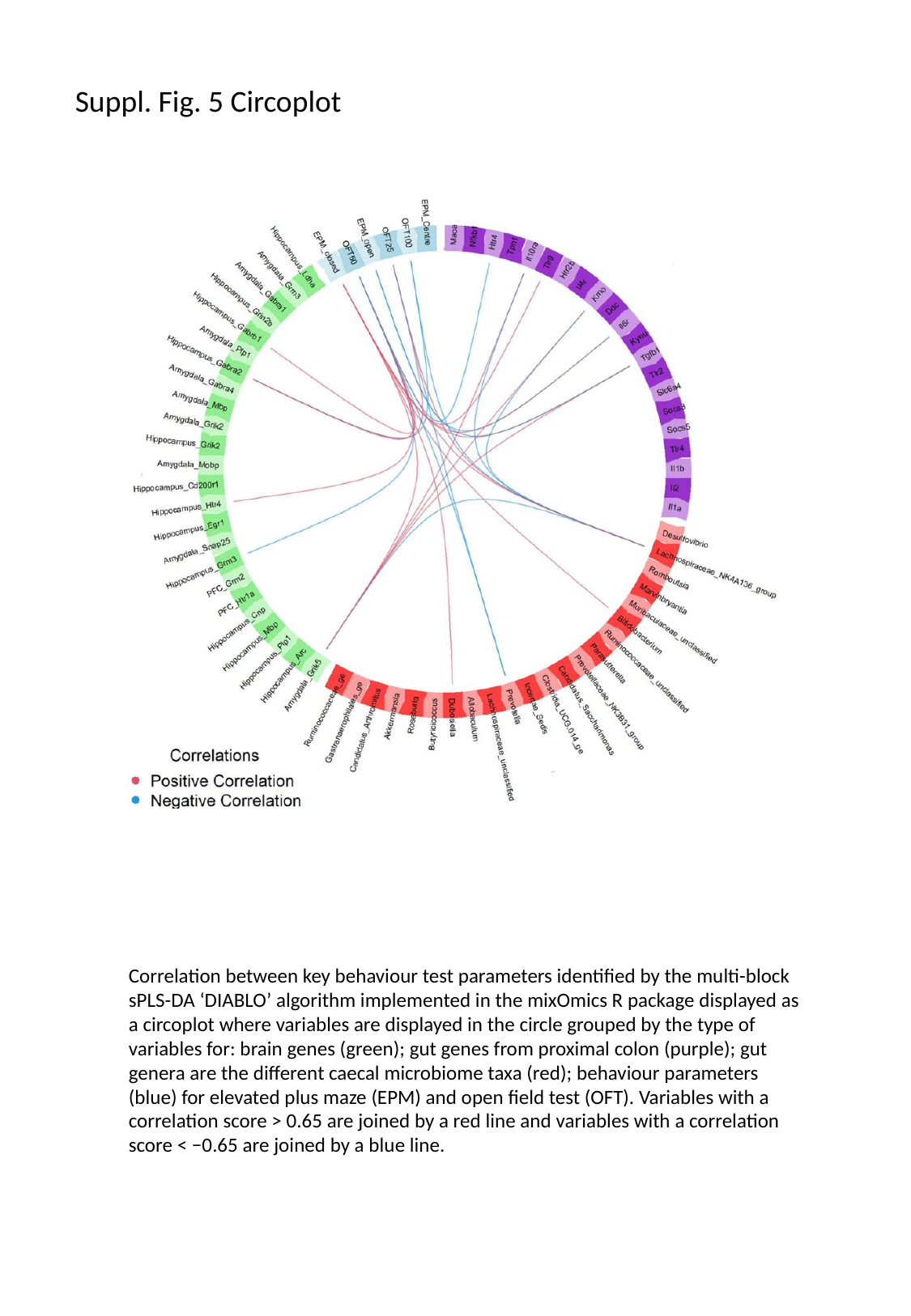

Suppl. Fig. 5 Circoplot
Correlation between key behaviour test parameters identified by the multi-block sPLS-DA ‘DIABLO’ algorithm implemented in the mixOmics R package displayed as a circoplot where variables are displayed in the circle grouped by the type of variables for: brain genes (green); gut genes from proximal colon (purple); gut genera are the different caecal microbiome taxa (red); behaviour parameters (blue) for elevated plus maze (EPM) and open field test (OFT). Variables with a correlation score > 0.65 are joined by a red line and variables with a correlation score < −0.65 are joined by a blue line.
